# Capture, mutual inhibition and release mechanism for aPKC–Par6 and its multisite polarity substrate Lgl

**DOI:** 10.1038/s41594-024-01425-0

**Published:** 2025-01-06

**Authors:** Christopher P. Earl, Mathias Cobbaut, André Barros-Carvalho, Marina E. Ivanova, David C. Briggs, Eurico Morais-de-Sá, Peter J. Parker, Neil Q. McDonald

**Affiliations:** 1 Signalling and Structural Biology Laboratory, Francis Crick Institute, London, UK; 2 Protein Phosphorylation Laboratory, Francis Crick Institute, London, UK; 3 Instituto de Biologia Molecular e Celular, Universidade do Porto, Porto, Portugal; 4 Instituto de Investigação e Inovação em Saúde (i3S), Universidade do Porto, Porto, Portugal; 5 Imperial College, London, UK; 6 School of Cancer and Pharmaceutical Sciences, King’s College London, Guy’s Campus, London, UK; 7 Institute of Structural and Molecular Biology, School of Natural Sciences, Birkbeck College, London, UK

**Keywords:** Cryoelectron microscopy, Kinases, Cell polarity

## Abstract

The mutually antagonistic relationship of atypical protein kinase C (aPKC) and partitioning-defective protein 6 (Par6) with the substrate lethal (2) giant larvae (Lgl) is essential for regulating polarity across many cell types. Although aPKC–Par6 phosphorylates Lgl at three serine sites to exclude it from the apical domain, aPKC–Par6 and Lgl paradoxically form a stable kinase–substrate complex, with conflicting roles proposed for Par6. We report the structure of human aPKCι–Par6α bound to full-length Llgl1, captured through an aPKCι docking site and a Par6^PDZ^ contact. This complex traps a phospho-S663 Llgl1 intermediate bridging between aPKC and Par6, impeding phosphorylation progression. Thus, aPKCι is effectively inhibited by Llgl1^pS663^ while Llgl1 is captured by aPKCι–Par6. Mutational disruption of the Lgl–aPKC interaction impedes complex assembly and Lgl phosphorylation, whereas disrupting the Lgl–Par6^PDZ^ contact promotes complex dissociation and Lgl phosphorylation. We demonstrate a Par6^PDZ^-regulated substrate capture-and-release model requiring binding by active Cdc42 and the apical partner Crumbs to drive complex disassembly. Our results suggest a mechanism for mutual regulation and spatial control of aPKC–Par6 and Lgl activities.

## Abstract (web summary)

Combining structural, biochemical, cellular and in vivo assays, the authors uncover the mechanism for capture and multisite phosphorylation of lethal (2) giant larvae by the atypical protein kinase C and partitioning-defective protein 6, revealing the basis for their mutual antagonism underpinning cell polarity.

## Main

Apical–basal polarity in epithelial cells is formed by the action of a conserved network of partitioning-defective (Par) proteins and their multiprotein complexes^1–3^. This network exhibits properties of mutual membrane exclusion (also known as mutual antagonism) and feedback to form polarized membrane domains with unique identities and sizes^4^. How these emergent and dynamic properties arise from the formation of multiprotein Par assemblies is not fully understood but phosphorylation of membrane-bound substrates is believed to be key. Within the apical membrane domain, the kinase–substrate relationships of the atypical protein kinase C (aPKC in *Drosophila*; two isoforms, aPKCι and aPKCζ, in human) dominate^5–9^. aPKC associates with the PDZ domain-containing protein Par6 to phosphorylate substrates such as Par1, Par2 and Par3, which frequently bear an FXR docking motif, driving them off apical membranes^10^. However, the precise contribution of Par6 in this process is unclear, with both activating and inhibitory roles toward aPKC kinase activity having been proposed^11,12^.

Polarity components oppose and repress aPKC activity in the cytoplasm or at lateral membranes restricting aPKC–Par6 activation to apical membranes^1,11^. A well-characterized example of a spatially controlled antagonist of aPKC is lethal (2) giant larvae (Lgl in *Drosophila*; Llgl1 and Llgl2 in mammals; in this study, we use Lgl generically across all species to refer to Lgl, Llgl1 and Llgl2, other than where we explicitly refer to Lgl in experiments in *Drosophila*). Lgl restrains aPKC activity except at the apical membrane where Lgl is removed directly by aPKC–Par6 through phosphorylation^1,11^. Lgl has a double β-propeller structure and contains multiple phosphorylation sites for aPKC–Par6, with at least three serine phospho-acceptor sites (S655, S659 and S663 in human Llgl1, within a segment defined hereafter as the P-site) that map within a key membrane-binding loop^13^. These highly conserved sites are required for efficient cortical displacement of Lgl but are both functionally and kinetically distinct^14–17^. Active apical aPKC suppresses apical action of Lgl by phosphorylating its P-site and displacing it from the membrane^18,19^. Lgl is consequently localized to the basolateral membrane in a steady state (together with Scribble and discs large) in a manner mutually exclusive with aPKC localization^18,20^. This reciprocal localization depends on Lgl phosphorylation by aPKC as an Lgl mutant lacking the three phosphorylation sites invades the apical membrane^14,20^. Paradoxically, Lgl forms a stable tripartite complex with aPKC–Par6 in a manner mutually exclusive with Par3, suggesting a more complex regulation than a simple hit-and-run phosphorylation mechanism^9,21^. The molecular mechanism for mutual inhibition is not understood despite its key role in many cell polarity contexts including epithelial polarity, asymmetric cell division and neuronal polarization.

To understand the antagonism between Lgl substrate and aPKC–Par6 kinase, the basis for multisite phosphorylation and how this stable three-way kinase–substrate complex is assembled, we determined a cryo-EM structure of an aPKC–Par6–Lgl tripartite complex. The structure reveals the intricate interplay between aPKC–Par6 and its substrate Lgl, which, together with in vitro and in vivo data, supports a capture-and-release mechanism involving a stable phospho-intermediate. In this mechanism, the Par6^PDZ^ domain is uncovered as the missing molecular link explaining exquisite substrate targeting of Lgl, the mutual inhibition of aPKC and its role as an apical sensor coupled to an allosteric release mechanism. Thus, we provide a description of a near-complete kinase–substrate multisite phosphorylation reaction cycle.

**Table 1:** Cryo-EM data collection, refinement and validation statistics

	aPKCι–Par6α–Llgl1 (EMD-18877), (PDB 8R3Y)
**Data collection and processing**	
Magnification	165,000
Voltage (kV)	300
Electron exposure (e^−^ per Å^2^)	48.1
Defocus range (μm)	−1.5 to −3.5
Pixel size (Å)	0.82
Symmetry imposed	*C1*
Initial particle images (no.)	1,069,057
Final particle images (no.)	121,194
Map resolution (Å)	3.6
FSC threshold	0.143
Map resolution range (Å)	10.6-2.0
**Refinement**	
Initial model used (PDB code)	6N8Q, 8R3X, 1NF3
Model resolution (Å)	3.58 (masked)
FSC threshold	0.5
Map sharpening *B* factor (Å^2^)	67.0
Model composition	
Nonhydrogen atoms	10,132
Protein residues	1,374
Ligands	1
*B* factors (Å^2^)	
Protein	52.95
Ligand	51.30
Root-mean-square deviations	
Bond lengths (Å)	0.024
Bond angles (°)	3.170
**Validation**	
MolProbity score	2.27
Clashscore	0
Poor rotamers (%)	3%
Ramachandran plot	
Favored (%)	92%
Allowed (%)	8%
Disallowed (%)	0%

FSC, Fourier shell correlation.

## Results

### Structure of a mutually antagonized polarity complex

**Fig. 1:**
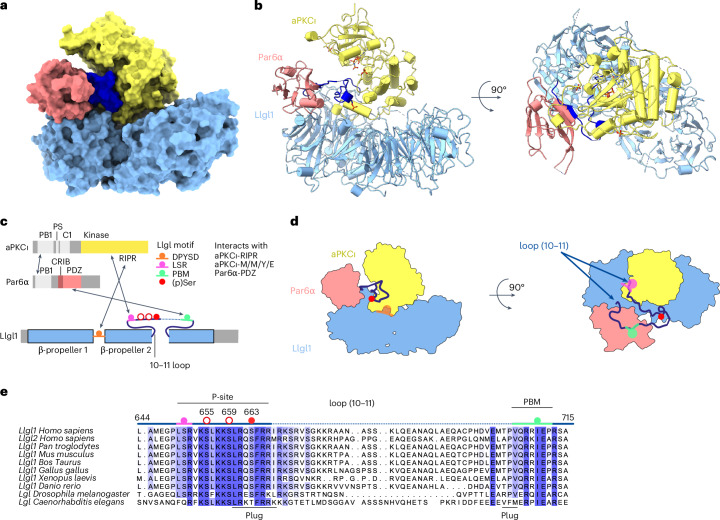
Cryo-EM structure of an antagonized aPKCι–Par6α–Llgl1 polarity complex. **a**, Surface rendering of the aPKCι–Par6α–Llgl1 cryo-EM structure. Surfaces for individual components are colored differently: Par6, salmon; aPKCι, yellow; Llgl1, light blue; Llgl1 loop (10–11) containing the P-site, navy. **b**, Ribbon diagram of the aPKCι–Par6α–Llgl1 complex, colored as in **a**. A stick representation for AMP-PNP is shown indicating the aPKCι nucleotide-binding site and the three phospho-residues aPKCι^pT412^, aPKCι^pT564^ and Llgl1^pS663^. **c**, Schematic of key interactions mapped onto the domain structures for each component. Grayed out segments indicate regions not defined in the final model. **d**, Schematized aPKCι–Par6α–Llgl1 structure showing two orthogonal slices through the structure with a similar view to **b**, mapping the approximate location of crucial interaction contacts and phospho-acceptor serine residues. **e**, Alignment of loop (10–11) sequences for Lgl homologs indicating conservation at opposing ends of the membrane-binding loop. The Lgl P-site and PBM are indicated. Phospho-acceptor sites and interaction contacts are colored as in **c**.

**Extended Data Fig. 1:**
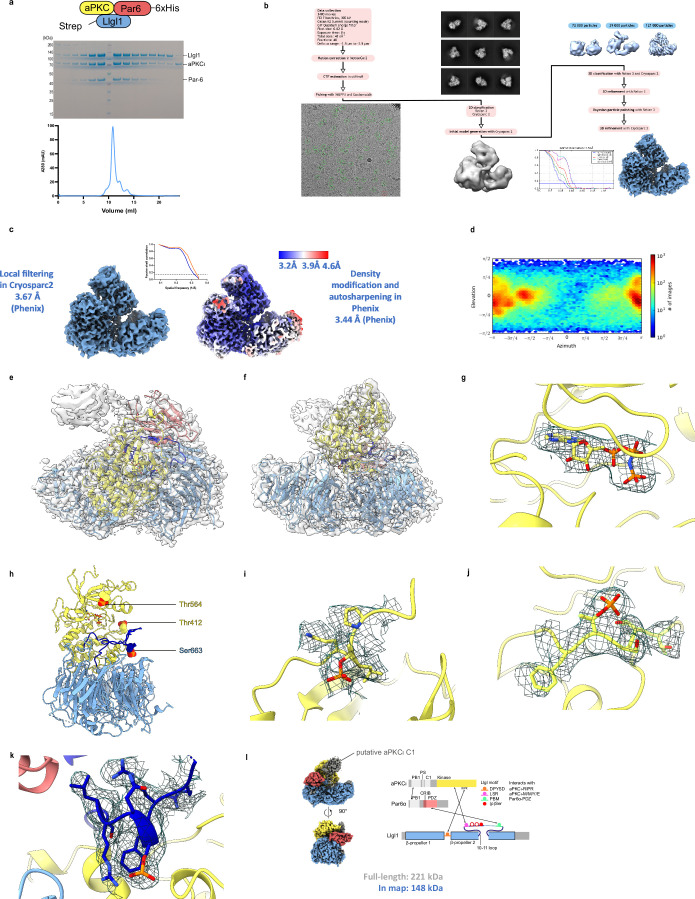
Purification and cryo-EM structure determination of an aPKCι-Par6α-Llgl1 complex. (**a**) Schematic of aPKCι-Par6α-Llgl1 complex and location of affinity purification tags. Representative SEC profile for aPKCι-Par6α-Llgl1 monitored by SDS-PAGE across gel filtration fractions. (**b**) Cryo-EM processing workflow for aPKCι-Par6α-Llgl1 complex. Data collection statistics are shown. 0.82 Å/pixel size was used and data were collected using a K2 detector on a Titan Krios microscope. A motion-corrected cryo-EM micrograph of the aPKCι-Par6α-Llgl1 complex is shown. The processing workflow shows a representative selection of cryo-EM 2D class-averages of aPKCι-Par6α-Llgl1 calculated using Cryosparc after using final 3D classification. Three 3D models were generated for which 121,000 particles contributed to the higher resolution 3D reconstruction. The structure was determined to an average resolution of 3.6 Å as judged by a Fourier-shell correlation (FSC = 0.143) criterion as shown. (**c**) Local resolution estimation. Shown are a local filtered map from CryoSparc2 (pale blue) and a density modified and auto-sharpened map from PHENIX indicating local resolution. Shown are FSC curves for the local filtered map (blue) at 3.67 Å and autosharpened map at 3.44 Å (red) calculated in Phenix. (**d**) Angular distribution of particles used in final reconstruction (**e**) Top view of model fitted to resolve-sharpened map contoured at 0.7 (**f**) Back view of model fitted to resolve-sharpened map contoured at 0.7 (**g**) Cryo-EM map density (dark blue chicken wire) superposed with final model within the aPKCι nucleotide pocket indicating bound AMP-PNP (**h**) Location of the three phospho-sites aPKCι ^pT412^, aPKCι^pT654^ and Llgl1^pS663^. Density for each modification is shown superposed with the final model for (**i**) the aPKCι turn motif pT654 residue, (**j**) aPKCι^pT412^ within the aPKCι activation loop and (**k**) the Llgl1^pS663^ residue within the P-site. (**l**) Regions not observed in the final structure (PB1 domains from aPKCι and Par6α, the pseudo-substrate and C1 domain of aPKCι) are greyed out, while poorer density proximal to the aPKCι N-lobe is not interpreted (dark grey density).

To obtain the structure of an aPKC–Par6–Lgl tripartite complex, we expressed and purified a complex containing human aPKCι, Par6α and Llgl1 from FreeStyle HEK293-F cells (Extended Data Fig. 1a). Formation of a soluble complex was confirmed by size-exclusion chromatography (SEC). The structure of the human aPKCι–Par6α–Llgl1 polarity complex was then determined at a nominal resolution of 3.44 Å using cryo-electron microscopy (cryo-EM) (Table 1 and Extended Data Fig. 1b–d). Maps were of sufficient quality to reliably dock the Par6^PDZ^ domain (Protein Data Bank (PDB) 1NF3), the aPKCι kinase domain (aPKCι^KD^; PDB 8RX3) and a crystal structure of full-length Llgl2 (PDB 6N8Q) with the readily recognizable double β-propeller (Fig. 1a,b and Extended Data Fig. 1e,f)^5,13,22^. After fitting each component and adjusting their sequences to the correct isoform, the molecular interfaces were rebuilt de novo, including all conserved parts of the previously unseen Llgl1 membrane-binding loop (10–11) harboring the P-site (Fig. 1c–e). This gave a reliable atomic model for the aPKCι–Par6α–Llgl1 tripartite assembly showing reciprocal interactions among all three components (Fig. 1b). The nucleotide pocket was occupied with adenylyl imidodiphosphate (AMP-PNP) added to the sample before grid preparation to stabilize the kinase core (Fig. 1b and Extended Data Fig. 1g). Two known phospho-threonine sites in aPKC (aPKCι^p^^T412^ and aPKCι ^pT564^) and one phospho-serine in Llgl1 (Llgl1^pS663^) were unequivocally identified from the cryo-EM map (Extended Data Fig. 1h–k). Regions toward the periphery of the cryo-EM map were less well resolved, including the pseudosubstrate membrane-binding element of aPKCι, as well as the PB1 domains of aPKCι and Par6α (Extended Data Fig. 1l). Additional density proximal to the αC-helix of the kinase domain N-lobe was observed in two-dimensional (2D) class averages and three-dimensional (3D) reconstructions (Extended Data Fig. 1l), which matched the overall shape and size of the aPKCι C1 domain but could not be fit reliably.

**Fig. 2:**
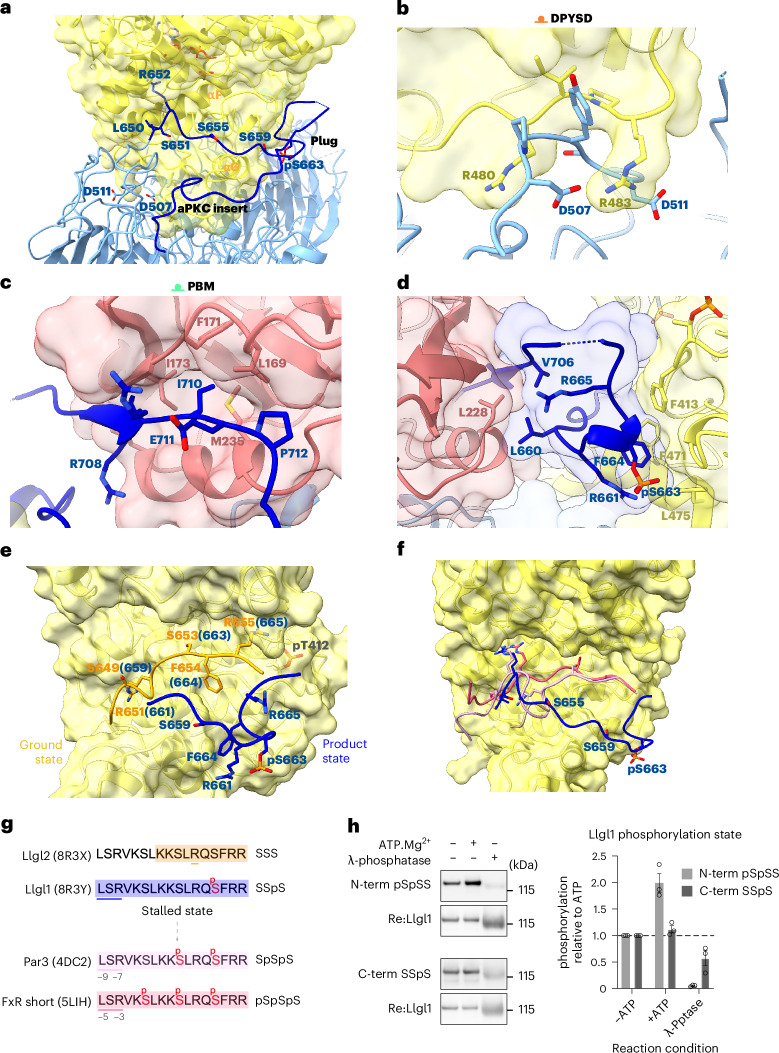
Capture of membrane-binding Llgl1 loop by aPKCι–Par6α prevents P-site phosphorylation progression. **a**, Extensive contacts between the aPKCι^KD^ (yellow solid rendering) and the Llgl1 loop (10–11) plug (navy). The location of the P-site phospho-acceptor sites in Llgl1 are indicated (S655, S659 and pS663), together with selected residues from the aPKCι LSR and RIPR motifs. The position of the plug domain formed around pS663 is also indicated. **b**, Close-up view of key residues from the RIPR motif contact within aPKCι and the reciprocal DPYSD motif in Llgl1. **c**, Close-up view of key residues from the Par6α PDZ contact with an internal PBM motif within the Llgl1 loop (10–11). **d**, Close-up view of the molecular plug formed by opposing ends of the Llgl1 loop (10–11), showing key residues close to the P-site sequence contributing to a small hydrophobic core or bridging between the aPKCι C-lobe pocket and the Par6α PDZ domain. **e**, Structural superposition of the Llgl2 P-site peptide crystal structure reported here overlaid with the trajectory of the phosphorylated product peptide from the tripartite cryo-EM structure. The superposition reflects the likely ground state of the unphosphorylated Lgl P-site and the conformational change induced upon phosphorylation of the initial pS663 site referred to as the stalled state. **f**, Proposed reconstruction of phosphorylation progression trajectory from the unphosphorylated state to the stalled state (pS663) to a double (pS659;pS663) then triple phosphorylation state (pS655;pS659;pS663). Previous structures have shown how substrates with an F^−9^XR^−7^ motif drive phospho-acceptor phosphorylation or through an F^−5^XR^−3^ motif (PDB 5LIH and 4DC2). The LSR motif of the Llgl1 and Llgl2 conformations adopts an identical pose to the FSR motif within the Par3 CR3 domain. We propose that release of the captured poise enables rotation of the P-site into the substrate-binding pocket for double (pS659;pS663) and subsequent triple phosphorylation (pS655;pS659;pS663). **g**, Summary of P-site phosphorylation progression highlighting the stalled state. Highlighted are the ordered P-site for Llgl2 (sand), the P-site from the cryo-EM structure (lilac) and modelled phosphorylation progression from published F^-9^XR^-7^ and F^-5^XR^-3^ peptide structures (pink/dark pink). Right hand side shows the phospho-status of the Lgl P-site. **h**, Immunoblot evidence that Llgl1 is substoichiometrically phosphorylated on the N-terminal serine residues but stoichiometrically phosphorylated on the C-terminal serine residue within the aPKC**ι**–Par6α–Llgl1 complex. Quantification is shown on the right (*n* = 3 independent in vitro assays, represented as the mean ± s.e.m.). Source data

The resulting aPKCι–Par6α–Llgl1 structure reveals the basis for coordinated capture of Llgl1 by Par6α and aPKCι, leading to a stably associated (trapped) phospho-intermediate of Llgl1. Multiple interactions stabilize the complex, centred on the second Llgl1 β-propeller and its central membrane-binding loop (10–11) that contains the P-site (Fig. 1c–e). The second β-propeller of Llgl1 makes the largest contact with an aPKC-isozyme specific sequence insert (residues G457–D468). This insert is located between the F and G helices of the kinase C-lobe extending away from the kinase to penetrate into the β-propellor cavity (Fig. 2a). This contact involves both acidic and hydrophobic contacts to bury a total surface area of ~2,374 Å^2^ (calculated at https://www.ebi.ac.uk/pdbe/pisa/), providing an extensive landing pad for the aPKCι^KD^. A second Llgl1–aPKCι^KD^ contact involves the aPKC RIPR motif (residues R480–R483) in the loop between the αG-helix and αH-helix, also at the base of the kinase C-lobe. This element straddles a complementarily charged DPYSD motif on Llgl1 located within loop (8–9) (Fig. 2b). This satisfyingly explains our previous observations that the RIPR motif is a necessary contact site for Llgl1/Llgl2 phosphorylation^23^. Both these contacts help orient the aPKCι^KD^ with its substrate-binding cleft facing toward and anchoring part of the ~72-aa-long membrane-binding loop (10–11) of Llgl1 containing the P-site. Residues L640-S641-R642 at the N terminus of the Llgl1 P-site engage a high-affinity docking site on aPKCι that was previously shown to be occupied by the Par3 FXR motif (Fig. 2a)^5^. The C terminus of the Llgl1 loop (10–11) is bound to the Par6α^PDZ^ domain through a previously unrecognized internal PDZ-binding motif (Llgl1^PBM^) spanning residues V706–P712 (Fig. 1e). The Llgl1^PBM^ is highly conserved in Lgl homologs, explaining the strong sequence constraints at the C-terminal side of the Llgl1 loop (10–11). The Llgl1^PBM^ adopts a short β-strand (Fig. 2c) that completes the central Par6^PDZ^ domain β-sheet as observed for other PDZ ligands^22^. Internal PBMs are poorly characterized but typically consist of a hydrophobic residue followed by an acidic residue (mimicking the C-terminal interaction of canonical PBMs)^24^. The key Llgl1 residue I710 occupies a hydrophobic pocket formed by L169, F171, I173 and M235 within the PDZ cleft and is followed by E711 (Fig. 2c). P712 inserts toward the PDZ core, disfavoring the PDZ conformation that binds C-terminal PBM motifs with high affinity, as discussed further.

Tethering of either end of the Llgl1 loop (10–11) to aPKCι^KD^ and Par6^PDZ^ as described above has three consequences. First, it separates the extremities of the loop and guides it into a deep cleft formed between aPKCι^KD^ and Par6^PDZ^ (Figs. 1a and 2a). Second, it brings two segments of the loop into close proximity to bridge between aPKCι^KD^ and Par6^PDZ^ by forming a molecular ‘plug’ domain nucleated around the single-site phosphorylation at Llgl1^pS663^ (Fig. 2d). The phospho-S663 site (Llgl1^pS663^) identified in the cryo-EM potential map forms a salt bridge with Llgl1^R661^, which packs against the Llgl1^F664^ side chain. These two residues in turn engage a conserved hydrophobic pocket within the aPKCι^KD^ C-lobe (involving residues F413, F471 and L475). The plug domain itself has a small hydrophobic core made up of L660 and V706 and the aliphatic parts of several basic residues (K658, R665 and R708) that cross from one side of the cleft to the other.

The third consequence of the tethered ends of the Llgl1 loop (10–11) is to trap the P-site in a nonproductive conformation for phosphorylation progression. By forming the plug domain around Llgl1^pS663^, the P-site effectively blocks access of S655 and S659 phospho-acceptor sites to the aPKCι substrate cleft and catalytic residues (Fig. 2e). The organization of this tripartite structure indicates that the aPKCι–Par6α heterodimer captures Llgl1, preventing its critical membrane interaction motif within the P-site from binding to the plasma membrane. Furthermore, the identification of this inhibited state further argues that additional steps must take place to promote multisite phosphorylation and in turn release Lgl from the complex.

### Evidence for an Lgl multisite phosphorylation trajectory

**Extended Data Fig. 2:**
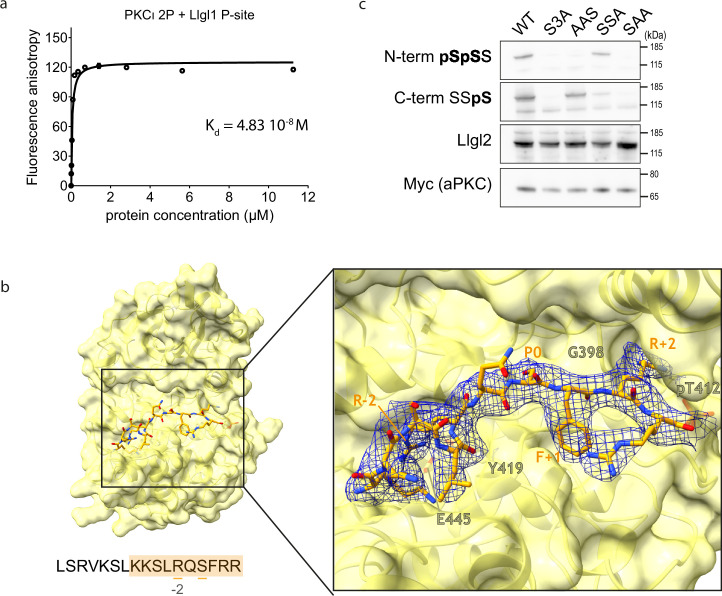
Multisite phosphorylation of Lgl. (**a**) Binding affinity of the Llgl1 P-site peptide to the aPKCι kinase domain (2P, phosphorylated at pT412 and pT564) measured by fluorescence anisotropy. (**b**) Crystal structure of the Llgl2 substrate peptide (sticks) bound to the aPKCι kinase domain (yellow surface) with the C-terminal Ser site positioned as the phospho-acceptor, residues are numbered from Ser at residue 0. Superposed is the final electron density map calculated using *sigma-A* weighted mFo-DFc coefficients. The ordered part of the Llgl2 P-site is shaded orange with the −2R and S phospho-acceptor site indicated by an underline. (**c**) Specificity of phospho-Llgl antibodies used in this study. Western blot analysis using phospho-specific antibodies against pS653 site or pS645/pS649 sites for different GFP-Llgl2 phospho-acceptor site serine mutations in the P-site of loop (10-11). Phosphorylation of WT and the indicated mutants of Llgl2 is shown upon co-expression with myc-tagged aPKCι. The western blot shows the specificity of the two different phospho-specific antibodies against either the pS653 site or pS645/pS649 sites (Llgl2). Representative western blot of n = 2 experiments. Source data

The observation that only Llgl1^S663^ in the P-site was phosphorylated within the cryo-EM structure agrees with previous in vitro peptide substrate studies that the C-terminal phosphorylation of the P-site is catalytically favored^16^ and presented first to aPKC. To support these findings, we investigated how the P-site binds to aPKC in its unphosphorylated state. A peptide spanning the Llgl1 P-site is able to bind the aPKCι^KD^ with an apparent affinity (*K*_d_) of 48 nM (Extended Data Fig. 2a). We screened for crystals of the Llgl1 and Llgl2 P-site peptides and determined a medium-resolution structure for the Llgl2 P-site peptide spanning residues 634–662 bound to aPKCι^KD^ in the absence of nucleotide. The Llgl2 P-site is identical to the Llgl1 sequence in this region but is ten residues shorter in numbering. The cocrystal structure of aPKCι^KD^–Llgl2^P-site^ revealed ordered contacts between residues 650 and 656 of the Lgl P-site, including the C-terminal S653 (equivalent to Llgl1 S663). In this poise, Llgl2^S653^ is bound ready for phospho-transfer, with Llgl2^F654^ in the hydrophobic +1 pocket (Fig. 2e and Extended Data Fig. 2b). The R655 side chain forms a hydrogen bond with the G398 carbonyl and a phosphate oxygen from phospho-T412, while the R651 side chain at the −2 position, relative to the phospho-acceptor, lies in a pocket formed between Y419 and E445 of aPKCι (Extended Data Fig. 2b). The structure confirms that the Lgl P-site binds aPKC, thereby presenting Llgl2^S653^ (equivalent to Llgl1^S663^) for initial phosphorylation, consistent with the cryo-EM structure. Absent from the X-ray structure is the L640-S641-R642 motif, suggesting that this motif may not be crucial for the initial C-terminal Llgl1 and Llgl2 phosphorylation. We propose that phosphorylation progression of the two N-terminal sites can be modeled from previous substrate peptide structures (PDB 5LIH and 4DC2), corresponding a short (F^−5^XR^−3^) or long (F^−9^XR^−7^) motif-binding mode, respectively (Fig. 2f)^5^. These modes differ in the register between the bound LSR motif and phospho-acceptor sites Llgl1 S655 and S659, which individually fit the F^−5^XR^−3^ and F^−9^XR^−7^ motifs. Superposition of previous substrate structures (PDB 4DC2 and 5LIH) indicate the precise conformational movement required to position the two N-terminal phospho-acceptor serine residues S655 and S659 close to the γ-phosphate of adenosine triphosphate (ATP). In each case, the phospho-acceptor Cα position would need a substantial conformational shift (of 9.7 Å and 13.4 Å, respectively) from the cryo-EM structure to be available for phospho-transfer. Thus, we can predict the phosphorylation trajectory from snapshots of unphosphorylated peptide (X-ray structure) to a stalled initial site phosphorylation (cryo-EM structure), as well as the S655 and S659 phosphorylation binding poses, on the basis of related substrate peptide structures (Fig. 2g).

Although the C-terminal P-site phosphorylation state (monophosphorylated at Llgl1^S663^) is the predominant form captured within the complex, further phosphorylation can be driven in vitro by adding ATP-Mg^2+^ to the complex. This increases the phosphorylation on the N-terminal S655 and S659 sites but not on the C-terminal S663 site as it is already stoichiometrically phosphorylated. This can be seen from immunoblots with antibodies specifically recognizing these phosphorylation sites (Extended Data Fig. 2c), supporting our structure of the single monophosphorylated state (Fig. 2h). Interestingly, previous in vivo studies in *Drosophila* showed that overexpression of the S656A;S660A double mutant (*Drosophila* Lgl numbering for the N-terminal sites) acts as a potent inhibitor of aPKC but did not clarify the underlying reason for the specific impact of this mutant version^17,25^. Phosphorylation at the available C-terminal serine in this double mutant would thus mimic the stalled phospho-intermediate we describe, effectively trapping aPKC–Par6–Lgl, thereby explaining the increased ability of this mutant to inhibit aPKC.

### Behavior of Lgl interface mutants in vitro

**Fig. 3:**
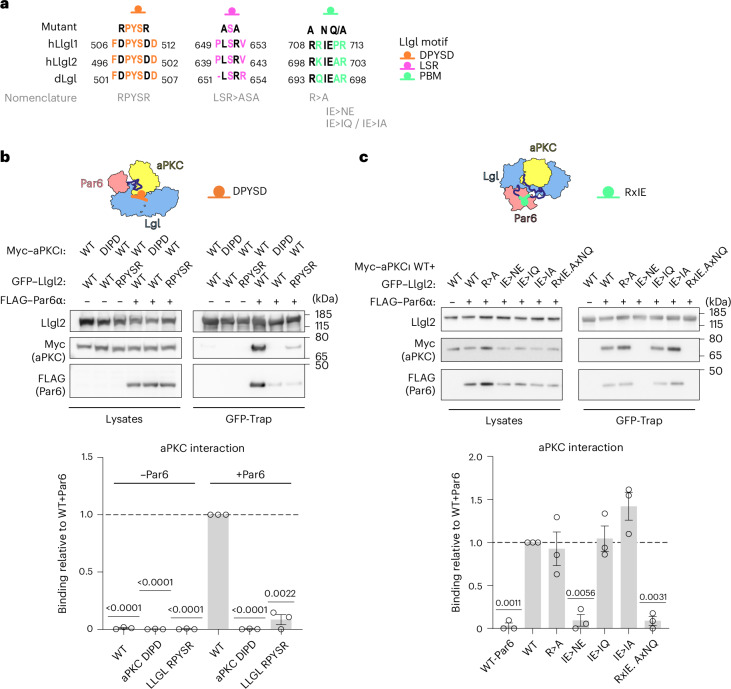
Impact of interface amino acid substitutions on aPKC–Par6–Lgl complex stability. **a**, Lgl-docking motifs within the complex and their conservation across isoforms and species. The mutants used to probe the function of each motif are indicated above and their nomenclature used throughout the manuscript is shown below in gray. **b**, Western blot analysis of GFP-Trap affinity pulldown assays from HEK293 cells expressing different GFP–Llgl2 docking interface mutants, Myc-tagged aPKCι and FLAG-tagged Par6α. Quantification of the western blots is shown directly below the blots (*n* = 3 biological replicates, represented as the mean ± s.e.m., analyzed using a two-tailed one-sample *t*-test). **c**, GFP-Trap affinity pulldown assays from HEK293 cells expressing selected GFP–Llgl2 mutants, Myc-tagged aPKCι and FLAG-tagged Par6α. Quantification of the western blots is shown directly below the blots (*n* = 3 biological replicates, represented as the mean ± s.e.m., analyzed using a two-tailed one-sample *t*-test). Source data

We then explored how different contacts within the complex contribute to stabilization of the stalled enzyme intermediate state. To do this, we prepared disruptive amino acid substitutions at the different conserved interfaces within Llgl2, a close human homolog to Llgl1 (ref. ^26^), whose structure^13^ and function^21,27,28^ are better characterized in cellulo. We interrogated interaction site mutants of Llgl2 expressed in HEK cells designed from the Llgl1 tripartite structure (Fig. 3a) by assessing their impact on the formation of the tripartite complex. Knowledge of the Llgl1 interaction site with the aPKCι RIPR motif allowed us to assess the impact of amino acid substitutions on either partner through engineering charge reversal substitutions to disrupt the Llgl2 interaction with the aPKCι RIPR motif. Consistent with our Llgl1-containing complex structure, we observed that formation of a stable complex between aPKCι and Llgl2 required coexpression with Par6α (Fig. 3b). When only aPKCι and Llgl2 were coexpressed, very low levels of aPKCι were recovered bound to GFP–Llgl2. Substituting the RIPR motif in aPKCι to DIPD (or a corresponding DPYSD>RPYSR substitution in Llgl2) suppressed the ability of Par6α to stabilize the tripartite complex, validating the structurally observed interface, in line with previous observations^23^. Evidence that the stability of the three-way complex is governed primarily by the Par6 contact is shown by substitution of the PBM residue in Llgl2 at I700 (hereafter Llgl2^IE>NE^) (Fig. 3a). Disrupting this key hydrophobic contact to the Par6^PDZ^ completely abolished the interaction with aPKCι and Par6α in HEK293 cells (Fig. 3c). These data indicate that formation of a stable complex in a steady state requires a three-way interaction involving an aPKC^KD^–Lgl contact (driven by the aPKCι RIPR motif) and crucially a Par6–Lgl contact (driven by the Par6^PDZ^ domain).

### Impact of Lgl interface mutants in polarized cells and in vivo

**Fig. 4:**
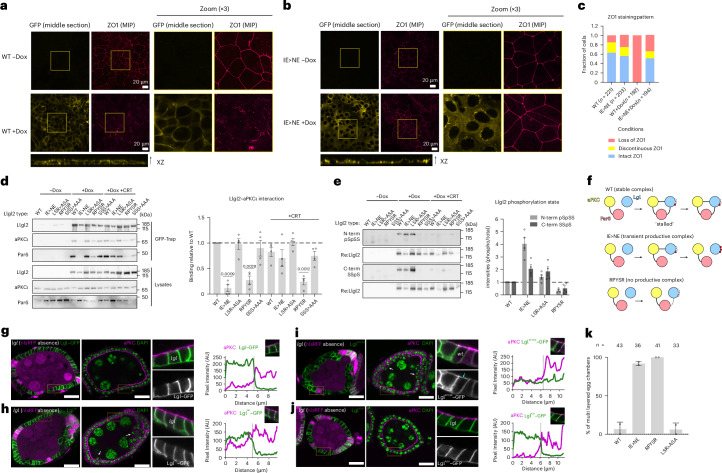
In cellulo and in vivo characterization of aPKC–Par6–Lgl complex interface mutants. **a**,**b**, Localization of WT (**a**) or Llgl2^IE>NE^ (**b**) in DLD1 cells and the effect on ectopic protein expression on DLD1 epithelial organization. WT or Llgl2^IE>NE^ was expressed in DLD1 cells by doxycycline induction. Llgl2 and ZO1 localization was followed using confocal microscopy. Representative micrographs are shown of one of three independent biological replicates. **c**, Quantification of cells in **a**,**b** with intact ZO1, discontinuous ZO1 or loss of ZO1. A representative experiment is shown of one of three biological replicates. **d**, Complex formation between Llgl2 and aPKCι–Par6. Cells expressing WT or mutant forms of GFP-tagged Llgl2 were lysed with or without pretreatment with the aPKCι inhibitor CRT0329868 and complex formation between Llgl2 and aPKCι–Par6 was followed by GFP-Trap (quantification on the right: *n* = 4 biological replicates, represented as the mean ± s.e.m., analyzed using a two-tailed one-sample *t*-test). The GFP-Trap Par6 signal was collected on a separate membrane to improve detectability. **e**, Phosphorylation state of ectopically expressed Llgl2 in DLD1 cells. Cells expressing WT or mutant forms of GFP-tagged Llgl2 were lysed with or without pretreatment with the aPKCι inhibitor CRT0329868 and phosphorylation of aPKCι target sites was followed using two antibodies, recognizing the two N-terminal and C-terminal phosphorylation sites of Llgl2 (quantification on the right: *n* = 4 biological replicates, represented as the mean ± s.e.m.). **f**, Proposed effects of amino acid substitutions in Llgl2 on its phosphorylation and complex formation with aPKCι. **g**–**j**, Confocal microscopy images of mosaic egg chambers of *lgl* mutant follicle cell clones (absence of nlsRFP, left panel), expressing the indicated Lgl versions in the *Drosophila* follicular epithelium and stained for DAPI (green, central panel) and aPKC (magenta, central panel). Close-up views and a plot of cortical pixel intensity from the basal side of the denoted region in follicle cells are shown on the right. AU, arbitrary units. **k**, Graph shows the frequency (mean ± s.d. of epithelial multilayering in egg chambers with mutant clones (larger than one quarter of the egg chambers) expressing the indicated Lgl versions (*n* is the number of egg chambers from two independent experiments). Source data

**Extended Data Fig. 3:**
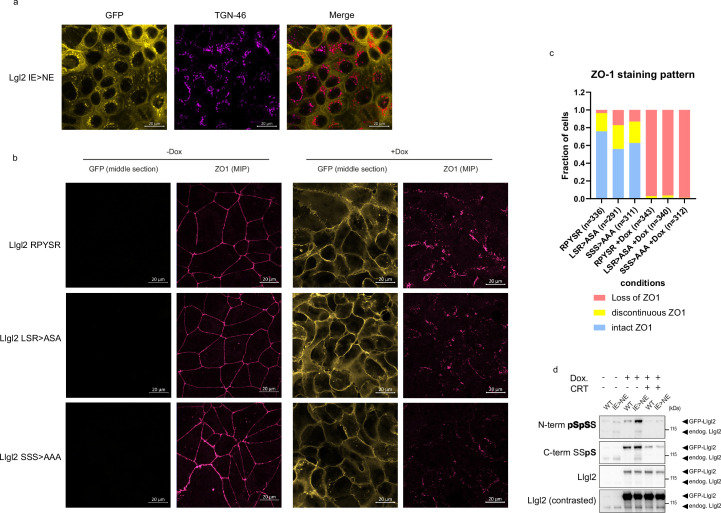
Properties of Llgl2 mutants in DLD1 cells. (**a**) Co-localization of Llgl-2^IE>NE^ in DLD1 cells with TGN-46. A subfraction of doxycycline (Dox)-induced Llgl-2^IE>NE^ protein localizes with foci staining positive for TGN-46. Representative micrographs shown from one of two separate cover slips (**b**) Localization of Llgl2 RPYSR, LSR > ASA, or SSS > AAA mutants of Lgl-2 in DLD1 cells and the effect of ectopic protein expression on ZO-1 staining as a marker of cell polarity. GFP-tagged WT or mutant forms of Llgl-2 were expressed in DLD1 cells via doxycycline induction. Llgl-2 and ZO-1 localization were monitored by confocal microscopy. Representative micrographs shown for one of three independent biological replicates (**c**) Quantification of ZO-1 staining patterns observed in (**b**). (**d**) Phosphorylation of endogenous Llgl1/2 with and without doxycycline induction. Overexpression of WT-Llgl2 suppresses the phosphorylation of endogenous Llgl1/2 in a dominant fashion, whereas the expression of Llgl-2^IE>NE^ has no effect. Source data

To explore the behavior of the validated interface mutants in the context of polarized cells, we expanded on a published phenotype in cultured epithelial cells, whereby wild-type (WT) Llgl2 overexpression causes a loss of polarity^21^. We engineered human DLD1 epithelial colorectal cancer cells to overexpress either WT GFP–Llgl2 or variants harboring interface substitutions that we characterized in the kinase-docking DPYSD motif (Llgl2^DPYSD>RPYSR^), the PBM motif (Llgl2^IE>NE^) and the substrate-docking LSR motif (Llgl2^LSR>ASA^). Each mutant expression was driven from a doxycycline-inducible promotor. In the absence of doxycycline, these cells displayed a largely polarized phenotype with intact zonula occludens 1 (ZO1) staining indicating properly formed tight junctions and polarized cell contacts (Fig. 4a,c). Inducing WT Llgl2 expression resulted in a dominant membrane localization of the overexpressed protein and loss of polarity, as evidenced by a complete loss of intact ZO1 staining (Fig. 4a,c). By contrast, when expression of the PBM Llgl2^IE>NE^ mutant was induced, no dominant phenotype was observed and levels of intact ZO1 staining were similar to the noninduced condition (Fig. 4b,c). Although a subfraction of the mutant protein localized to the plasma membrane, the majority remained in the cytoplasm and notably also clustered in foci that stained positive for the *trans*-Golgi marker TGN46 (Extended Data Fig. 3a). Llgl2 was initially shown to localize at the Golgi and the localization of this mutant may, therefore, reflect a distinct state of this protein^19^. By contrast, overexpressing the KD-contacting Llgl2^DPYSD>RPYSR^ or Llgl2^LSR>ASA^ variants or a nonphosphorylatable triple serine-to-alanine mutant (Llgl2^SSS>AAA^) resulted in a similar phenotype to that seen with overexpressed WT Llgl2, with a predominant membrane localization and loss of intact ZO1 staining (Extended Data Fig. 3b,c).

In parallel with these observations, we studied tripartite complex formation and Llgl2 phosphorylation in a steady state in these cells. The WT Llgl2 protein formed a stable complex with aPKCι and Par6, similar to a nonphosphorylatable Llgl2^SSS>AAA^ mutant. This indicates that overexpressed Llgl2 accumulates endogenous aPKCι–Par6 into a stalled complex (Fig. 4d). The Llgl2^IE>NE^ mutant on the other hand did not form a stable complex with aPKCι–Par6 in a steady state in the DLD1 cells (Fig. 4d), consistent with our observations in HEK293 cells. However, compared to the WT protein, the PBM mutant Llgl2^IE>NE^ showed increased phosphorylation, predominantly at the N-terminal serine sites (Fig. 4e). This observation argues that the Llgl2^IE>NE^ protein only forms a transient phosphorylation complex with aPKCι, whereas the stable interaction of WT Llgl2 with Par6 suppresses catalytic activity, mainly at the N-terminal S645 and S649 phosphorylation sites. Additionally, the transient interaction of the Llgl2^IE>NE^ mutant allows for the phosphorylation of endogenous Llgl1 and Llgl2 by aPKC–Par6, while the overexpressed WT mutant suppresses this (Extended Data Fig. 3d). The third mutant Llgl2^LSR>ASA^ displayed slightly elevated levels of phosphorylation skewed toward its C-terminal phosphorylation site, while exhibiting WT levels of N-terminal serine phosphorylation and tripartite complex formation (Fig. 4d,e). The fact that this substitution results in higher relative levels of C-terminally phosphorylated Llgl2 argues that there is an increased turnover rate compared to the WT protein and supports the notion that the LSR motif aids phosphorylation of the N-terminal S645 and S649 sites but appears dispensable under these conditions. The Llgl2^DPYSD>RPYSR^ mutant breaks the aPKCι docking contact, leading to reduced Llgl2 binding to aPKCι and reduced levels of phosphorylation (Fig. 4d,e). In contrast to the HEK293 cells and in the context of coexpression with Par6α, the loss of the aPKC–Llgl2 contact is less disruptive to the three-way complex in DLD1 polarized cells, with residual binding likely mediated by Par6. This is supported by the observation that treatment with an aPKC-selective chemical inhibitor CRT0329868 (ref. ^29^) trapped the PBM-defective Llgl2^IE>NE^ variant in the tripartite complex, whereas it had no such effect on the kinase contact Llgl2^DPYSD>RPYSR^ variant (Fig. 4d), highlighting that binding to the KD was impaired in the latter case and not in the former.

Taken together, these data suggest that overexpressed WT Llgl2 forms stable complexes with aPKCι–Par6, trapping it in an autoinhibited complex with low turnover (Fig. 4f, top row). The PBM mutant Llgl2^IE>NE^ instead forms a transient complex with aPKCι that is efficiently phosphorylated and released, resulting in a higher stoichiometry of phosphorylation and cytosolic protein that accumulates in the Golgi compartment (Fig. 4f, middle row). The kinase-docking Llgl2^DPYSD>RPYSR^ variant, in contrast, displays reduced levels of complex formation in the DLD1 cells and reduced capacity to form a productive enzyme–substrate complex with aPKCι–Par6, resulting in reduced phosphorylation (Fig. 4f, bottom row). Produced in excess, this mutant can disrupt polarity likely because it shows residual binding to aPKC and cannot be cleared from membranes by phosphorylation, resulting in an increased pool of membrane-bound Llgl2 acting on aPKC and other downstream effectors such as myosin II (ref. ^30^).

To characterize these Lgl mutants in vivo, we investigated whether these Lgl mutants could support epithelial apical–basal polarity in the monolayered follicular epithelium of the *Drosophila* ovary. This is a well-defined system to probe the in vivo role of apical–basal polarity proteins such as Lgl, for which loss-of-function alleles induce the formation of a multilayered epithelium^31,32^. We performed rescue experiments in mosaic tissue containing *lgl*-null follicle cell clones to analyze the ability of the aforementioned interface mutants to sustain apical–basal organization (mutants defined in Fig. 3a). The Lgl variants were tagged with a C-terminal GFP tag and expression was driven specifically in the follicular epithelium using the upstream activation sequence (UAS)–Gal4 system^33^. We then imaged fixed mosaic *Drosophila* egg chambers costained for aPKC where the localization of each Lgl variant was detected by the GFP signal and where *lgl*-null cells were identified by the absence of nlsRFP (red fluorescent protein with a nuclear localization sequence). Expressing the GFP-tagged versions of WT Lgl or the Lgl^LSR>ASA^ mutant restored a monolayered architecture in the absence of endogenous Lgl (Fig. 4g,j,k), whereas the kinase-docking mutant Lgl^DPYSD>RPYSR^ and the PBM mutant Lgl^IE>NE^ showed a large frequency of egg chambers with the multilayered phenotype (Fig. 4h,i,k).

The finding that the Lgl^DPYSD>RPYSR^ and the Lgl^IE>NE^ mutants are unable to support intact tissue polarity indicates the importance of regulated aPKC–Par6–Lgl complex assembly and disassembly to support apical–basal polarity. However, these mutants have distinct functional and localization properties. First, the PBM mutant Lgl^IE>NE^ retains some ability to sustain apical–basal polarization as indicated by the polarized enrichment of aPKC in monolayered patches that lack endogenous Lgl but express Lgl^IE>NE^ (Fig. 4h, close-up view). In contrast, the mutant disrupting the KD contacts, Lgl^DPYSD>RPYSR^, is unable to support polarity. Second, whereas Lgl^IE>NE^ is cleared from the apical compartment and restricted to the basolateral cortex in polarized cells (Fig. 4i, panel C), the Lgl^DPYSD>RPYSR^ mutant distributes all over the cell, invading the apical domain even in control nlsRFP-positive cells (Fig. 4j, close-up view). Apical invasion by the Lgl^DPYSD>RPYSR^ mutant is consistent with the lack of Lgl phosphorylation because it cannot form a productive tripartite complex with aPKC–Par6 promoting its removal. Taken together, these data stress the in vivo importance of the stability of the aPKC–Par6–Lgl complex and the dynamics of complex turnover in relation to the phenotype. Inappropriate early release of the Par6^PDZ^ domain in the Lgl^IE>NE^ mutant likely promotes untimely complex disassembly and reduces the ability of Lgl to antagonize aPKC to maintain apical–basal organization.

### Cdc42 and Crb trigger ATP exchange and complex disassembly

**Fig. 5:**
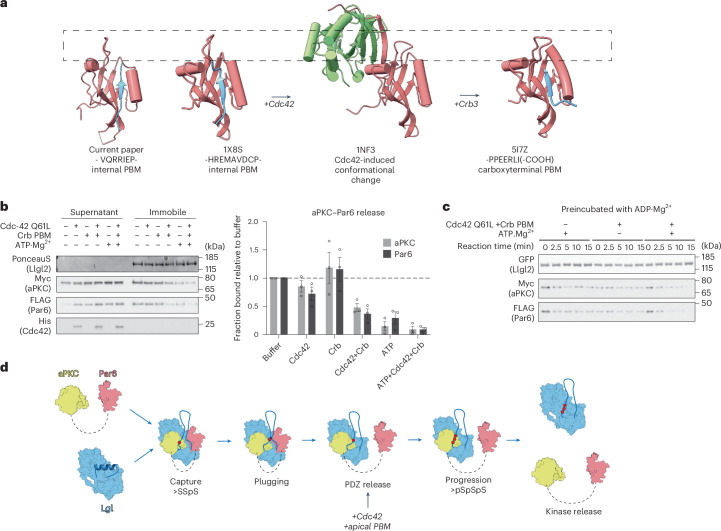
Mechanism of aPKC–Par6–Lgl complex disassembly. **a**, Conformational differences between PDZ domains bound to C-terminal or internal PBM ligands and the conformational change triggered by GTP-bound Cdc42 binding. PDZ domains, salmon; PBM ligands, blue; Cdc42, green. A dashed box is shown to emphasize the carboxylate-binding loop conformation. **b**, Complex dissolution in vitro monitored by western blot in the presence of the indicated factors. Quantification of the western blot is shown on the right (*n* = 3 biological replicates, represented as the mean ± s.e.m.). **c**, Time course of complex disassembly when preincubated with ADP-Mg^2+^. Kinetics are shown in Extended Data Fig. 4b,c (*n* = 4 biological replicates, represented as the mean ± s.e.m.). **d**, Model for a full phosphorylation cycle for Lgl driven by aPKC–Par6 integrating the findings reported here. Lgl is captured through the DPYSD motif interaction with the aPKC^KD^ RIPR motif. The complex is stabilized through the Par6^PDZ^–Lgl^PBM^ contact and an initial phosphorylation event at the C-terminal serine residue setting up the formation of the plug domain. Further phosphorylation in this stable complex is proposed to be inefficient as the P-site is rotated away from the active site and stabilized by the molecular plug. When the PDZ contact is released by Cdc42–GTP and by a competing PBM-containing protein, the Lgl P-site can be efficiently phosphorylated, leading to kinase release. Source data

**Extended Data Fig. 4:**
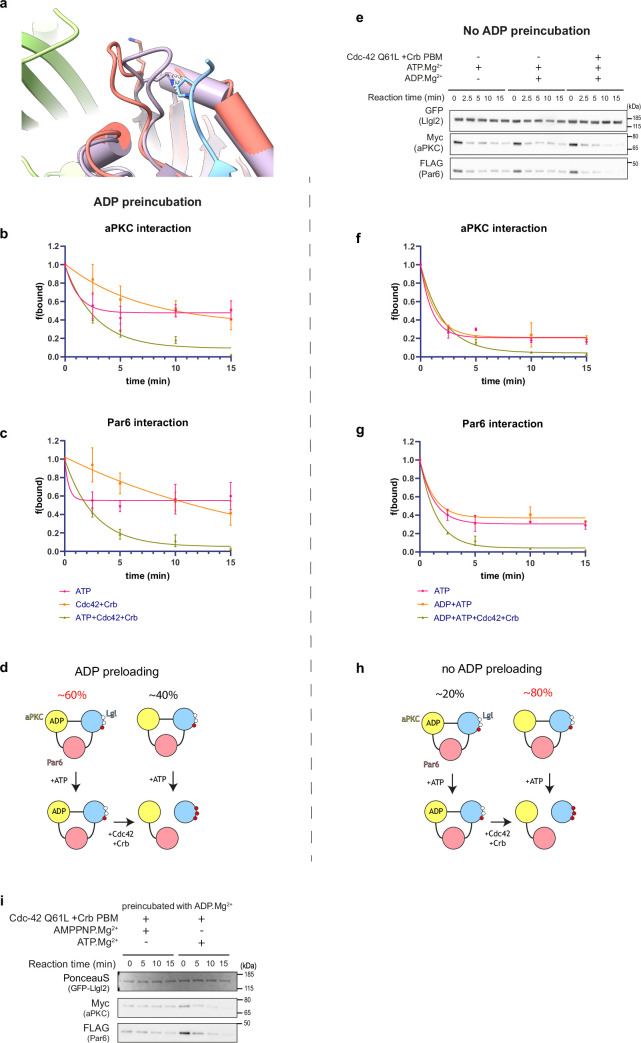
Release of Lgl from aPKC-Par6 is driven by Cdc42, Crb and ADP-ATP exchange. (**a**) Steric clash between K162 (human Par6α numbering) of the Par6 PDZ domain in the Cdc42-induced conformation (5I7Z) shown in purple and P712 from a structural superposition of the Llgl1 internal PBM (this study) shown in blue. The clash indicates that the Cdc42-induced conformation is incompatible with a bound internal PBM. Cdc42 is coloured in green and the Par6α PDZ domain of the reported structure in salmon. (**b**) aPKCι release kinetics quantified from the aPKCι-Par6α-Llgl2 complex western blots as shown in Fig. 5c. Quantified from n = 4 biological replicates represented as mean ± SEM (**c**) Par6 release kinetics shown in Fig. 5c. Quantified from n = 4 biological replicates represented as mean ± SEM. Points fitted to a one phase exponential decay dissociation curve using GraphPad Prism (**d**) Schematic representation of the predicted proportions of tripartite complex with aPKCι in the apo or ADP-bound state when preloaded with ADP.Mg^2+^ and their respective trajectories for release. Proportions inferred from residually bound protein levels in panels b and c. (**e**) Time course of aPKCι-Par6 release from Llgl2 and the influence of the indicated factors without preincubation with ADP.Mg^2+^. Representative western blot of n = 2 biological replicates. (**f**) aPKCι release kinetics quantified from western blots and shown in e. Quantified from n = 2 biological replicates represented as mean ± SEM (**g**) Par6 release kinetics as in e. Quantified from n = 2 biological replicates represented as mean ± SEM. Points fitted to a one-phase exponential decay dissociation curve using GraphPad Prism (**h**) Schematic representation of the predicted proportions of complex obtained as apo or ADP-bound and their respective trajectories for release. Proportions inferred from residually bound protein levels in panels f and g. (**i**) Time course of complex disassembly induced by ATP.Mg^2+^ versus AMP-PNP.Mg^2+^ when pre-incubated with ADP-Mg^2+^. Representative western blot of n = 3 biological replicates. Source data

Having established that the Par6^PDZ^ engagement is crucial for trapping the complex, we investigated possible triggers for its release. A prime candidate is Cdc42–GTP (guanosine triphosphate), which has been shown to induce a conformational switch in Par6^PDZ^ to alter its specificity for C-terminal PBM partners^22,34,35^ (Fig. 5a). However, the biological importance and context for this allosteric switch mechanism have not been demonstrated. Comparison of the PDZ domain conformation bound to the internal Llgl1 PBM showed close similarity to the previously reported complex with an internal Pals1 PBM ligand^22^ (Fig. 5a). However, comparison to the Crumbs3 (Crb3) C-terminal PBM bound complex revealed a steric clash between P712 of Llgl1 (proceeding the PBM at I710-E711) and a lysine in Par6 (K165 in *Drosophila* Par6, equivalent to K162 in human Par6α). This lysine is part of an allosteric dipeptide switch motif (L164-K165 in *Drosophila* Par6 and H161-K162 in human Par6α) that was proposed to bind Crb3 with high affinity in the presence of Cdc42–GTP^35^ (Extended Data Fig. 4a). Furthermore, the overall conformation of the carboxylate-binding loop between β-strands 1 and 2 of the PDZ domain also contributes to the affinity switch by sterically hindering internal PBM ligands. We, therefore, considered whether Cdc42–GTP could act as a trigger to release Par6^PDZ^ within the aPKC–Par6–Lgl complex, enabling an apical PBM partner such as Crumbs to bind Par6 (Fig. 5a). Consistent with this, a study by Dong et al. indeed proposed that Crumbs was required to switch Par6 from an inhibitory role to an activating role^11^.

To test these ideas, we coexpressed aPKCι–Par6α together with GFP–Llgl2 in cells, immobilized the tripartite complex on GFP-Trap beads and then added recombinant Cdc42–GTPγS with a Q61L mutant preventing GTP hydrolysis and/or the Crb3 PBM peptide. Comparing the residual bound protein fraction, we observed that the stability of the aPKCι–Par6α–Llgl2 complex was compromised by Cdc42–GTP but also required Crb3 PBM, leading to a 50% reduction in aPKCι–Par6α binding after 20 min (Fig. 5b). ATP alone also disassembled the complex, presumably by driving phosphorylation to completion (Figs. 5b and 2g). Adding both Cdc42–Crb3 and ATP led to an even more efficient release of aPKCι–Par6α with very low residual binding remaining after 20 min. These data indicate that Cdc42 and the Crb3 PBM can destabilize the tripartite complex and that phosphorylation drives full dissociation. We, therefore, wondered whether Cdc42-mediated PBM release impacts the ATP-driven release of aPKC–Par6α. To probe this effect, we followed complex dissociation kinetics with ATP and/or Cdc42–Crb3 at 16 °C (Fig. 5c). In these assays, the complex was preloaded with adenosine diphosphate (ADP) to reflect the product state of the complex after the first reaction step. ATP or Cdc42–Crb3 PBM alone dissociated only a small fraction of the complex under these reaction conditions (Fig. 5c and Extended Data Fig. 4b–d). Adding both Cdc42–Crb3 PBM and ATP to the ADP-preloaded complex resulted in a full release of aPKCι–Par6α after 15 min. Importantly when the complex was not preloaded with ADP, ATP itself was sufficient to drive a large proportion of aPKCι–Par6α release, which occurred very rapidly after addition, with about 20% of the complex remaining intact after 2.5 min (Extended Data Fig. 4e–g). This indicates that, in the apo state of the complex obtained during immunoprecipitation (~80%), ATP loading, phosphorylation and release are efficient, whereas, in the ADP-bound state (~20%), reflecting the likely nucleotide pocket status of the stalled complex within the cell, these steps require both Cdc42 and Crb3 (Extended Data Fig. 4e–h). The reaction rate is, thus, limited by ADP release and we conclude that Cdc42–Crb3 PBM binding to the Par6^PDZ^ enhances this rate-limiting nucleotide release step. The ATP-bound conformation in itself is not sufficient to drive release, as AMP-PNP loading does not result in the release of aPKC–Par6 (Extended Data Fig. 4i). Furthermore, the cryo-EM structure was prepared with AMP-PNP, indicating that processive phosphorylation is required for release.

## Discussion

### Mechanistic model for Lgl capture by aPKC–Par6, mutual inhibition and release

**Extended Data Fig. 5:**
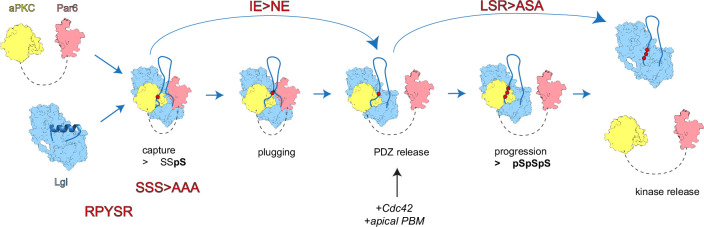
Impact of disruptive Lgl mutations on tripartite complex behaviours. Explanation of the behaviours and properties of four disruptive Lgl mutations described in the text, referring to the overall model presented in Fig. 5. The IE > NE mutant in the PBM bypasses the plugged state of the complex, leading to rapid phosphorylation progression and complex dissolution. The LSR > ASA mutant of the high-affinity kinase docking motif is less stably tethered to the kinase domain as the wild-type protein, resulting in more rapid release combined with less efficient N-terminal Ser phosphorylation, while C-terminal phosphorylation is unaffected. The RPYSR mutant displays a decreased kinase domain interaction and is unable to correctly position the kinase domain for efficient phospho-transfer, resulting in an overall suppression of phosphorylation. The SSS > AAA non-phosphorylatable mutant is trapped in the capture state as it cannot be phosphorylated to form the initial product state required to assemble the plug. It behaves similar to the WT protein in overexpressed conditions, which is trapped in the plugged state, as it likely saturates the endogenous release mechanism impeding the progression of the reaction.

Using cryo-EM, biochemical, cellular and in vivo experiments, we identified and validated key interaction sites and their functional roles within the tripartite aPKC–Par6–Lgl complex, supporting an integrated mechanistic model of complex assembly, mutual inhibition and dissolution. Our data reveal how Lgl is initially captured and oriented in a coordinated fashion by aPKC and Par6 (Fig. 5d). The aPKC kinase domain docks onto the second β-propeller of Lgl using a previously identified RIPR docking motif and an aPKC-isozyme-specific insert^23^. The complex is held together by the Par6^PDZ^ domain interaction with the C-terminal part of the Lgl (10–11) loop harboring an internal PBM motif. A single C-terminal P-site phosphorylation promotes contacts with aPKC^KD^ proximal to the activation loop in the C-lobe, driving formation of a molecular plug that bridges aPKC and Par6. This plug maintains the N-terminal phosphorylation sites oriented away from the substrate cleft and catalytic site. In this complexed and inhibited state, phosphorylation progression is inefficient as the P-site and PBM motifs are tethered, effectively stalling phosphorylation. When the Par6^PDZ^ domain dissociates to release the Lgl^PBM^, an event that can be triggered by a Cdc42-dependent conformational change (assisted by an apical protein with a high affinity C-terminal PBM), Lgl P-site phosphorylation is allowed to progress. This model explains why interface substitutions that block aPKCι docking with Lgl prevent phosphorylation and disrupt monolayer organization and polarity in vivo, whereas substitutions that perturb Par6^PDZ^ domain capture of the Lgl protein also do not support maintenance of normal apical–basal organization in vivo yet substantially enhance Lgl phosphorylation (Extended Data Fig. 5).

Several models, as previously reviewed^1^, have proposed that the aPKC–Par6 complex antagonizes Lgl by displacing it from the apical membrane by Cdc42-induced phosphorylation of its membrane-binding loop, consistent with the structure reported here. How then does Lgl reciprocally antagonize aPKC in the bound state? Our structure suggests that disassembly of the tripartite complex requires both multisite phosphorylation of the Lgl P-site and release of the Par6^PDZ^ from Lgl. When the Par6^PDZ^ domain is engaged, we find that the progression of P-site multisite phosphorylation is impeded. Lgl, thus, effectively antagonizes aPKC–Par6 by trapping it in a tethered intermediate product state. We propose that the controlled release of Par6 under normal circumstances arises through a Cdc42-induced conformational change and/or competition with binding partners of Par6 at the apical membrane, such as Crumbs^11,36,37^, leading to plug domain dissolution, rapid ADP-to-ATP exchange, progression of phosphorylation and disassembly of the tripartite complex. An important implication of this model is that, if Lgl encounters aPKC at the basolateral membrane, it inhibits kinase activity and escorts aPKC away. Such a repressed tripartite aPKC–Par6–Llgl complex would, therefore, be able to ‘sense’ the apical membrane compartment that contains multiple (competing) partners of Par6 and could respond accordingly unleashing aPKC–Par6 catalytic activity. Our model can also explain the apparently conflicting roles of Par6 as an auxiliary subunit of aPKC, where it is able both to activate and to repress aPKC activity^11,12^, in addition to contributing to substrate targeting of Lgl.

The precedent set by the existence of the stalled intermediate state that we describe here, as well as the requirement for additional regulatory inputs to facilitate nucleotide exchange, has broader implications for kinases. There are numerous examples of ‘processive’ phosphorylation events in nature, as previously reviewed^38^, and it will be of interest to understand whether some of these examples are also subject to intermediate product phospho-states requiring regulatory inputs.

In summary, we conclude that the tripartite aPKCι–Par6α–Llgl1 complex that we characterized structurally and functionally reflects the first cycle or step of the kinase reaction, with Llgl1 monophosphorylated and an internal Llgl1^PBM^–Par6^PDZ^ interaction that precludes further phosphorylation, stalling processivity. Mutational interrogation of the interfaces that define this complex confirmed their critical role in determining polarity. Completion of the reaction cycle based on mutagenesis and reconstitution experiments demonstrated that the subsequent engagement of Cdc42–GTP, switching the Par6^PDZ^ domain to engage with a Crumbs C-terminal PBM, promotes nucleotide exchange, creating an efficient ATP-dependent completion of phosphorylation and dissociation of the complex. These unprecedented properties revealed through structural analysis provide a comprehensive view of the mutual inhibition of aPKC–Par6 and Lgl, as well as the regulatory inputs that determine the dynamics of their engagement, and set a series of important precedents informing kinase–substrate relationships.

## Methods

### Cell lines and reagents

HEK293T cells were grown in DMEM supplemented with 10% (v/v) FBS (Thermo Fisher Scientific), 100 U per ml penicillin and 100 µg ml^−1^ Streptomycin (Thermo Fisher Scientific). FreeStyle 293-F cells were grown in FreeStyle 293 expression medium (Thermo Fisher Scientific). DLD1-FlpIn-TREx cells were grown in DMEM supplemented with 10% (v/v) FBS (Thermo Fisher Scientific), 100 U per ml penicillin and 100 µg ml^−1^ streptomycin (Thermo Fisher Scientific). DLD1-FlpIn-TREx cells stably harboring genes for WT and mutant forms of GFP–Llgl2 were selected and grown in the same medium supplemented with 500 µg ml^−1^ hygromycin B. Transcription of the transgenes was induced with 200 ng ml^−1^ doxycycline for 16 h unless stated otherwise. Unless stated otherwise, all cloning enzymes were purchased from New England Biolabs (NEB) and other chemicals were purchased from Sigma-Aldrich. GFP-Trap agarose was from Chromotek. Anti-Myc (9B11), anti-GFP (4B10), anti-His (rabbit), anti-phospho-Llgl1/Llgl2 S663, secondary HRP-linked goat anti-rabbit and horse anti-mouse antibodies were from Cell Signaling Technologies. Anti-FLAG M2 antibody was from Sigma-Aldrich, anti-phospho-Llgl1/Llgl2 S650/S654 antibody was from Abgent, anti-Llgl1 monoclonal antibody was from Abnova, anti-Llgl2 antibody was from Abcam, anti-Par6B (B-10) antibody was from Santa Cruz, anti-TJP1 antibody was from Atlas and anti-TGN46 antibody was from Abcam. Polyethyleneimine (PEI) was from Polysciences. Mutagenesis and cloning were performed using In-Fusion (Takara) or Gibson assembly (NEB). Plasmids and primers used in this study are listed in Supplementary Table 1. Peptides used were made in-house.

### Protein expression and purification

pCDNA3.1+ plasmids were modified to contain N-terminal tobacco etch virus (TEV) protease-cleavable Twin-Strep or 6xHis tags. Genes for full-length aPKCι, Par6α and Llgl1 were amplified by PCR from human complementary DNA and inserted into the modified plasmids by Gibson assembly to produce Twin-Strep-tagged Llgl1 and 6xHis-tagged Par6α. FreeStyle 293-F cells (Thermo Fisher Scientific) were transfected with expression plasmids using linear PEI (molecular weight, 25,000; Polysciences). Cells were harvested by centrifugation after 5 days of shaking at 120 rpm in 8% CO_2_ at 37 °C. Cell pellets were resuspended in buffer A (20 mM HEPES pH 7.5, 150 mM NaCl and 0.5 mM TCEP) supplemented with cOmplete EDTA-free protease inhibitor cocktail tablets (Roche) and lysed by sonication. Cell lysates were clarified by centrifugation and incubated with StrepTactin XT Sepharose (GE Healthcare) for 90 min at 4 °C. After extensive washing, bound proteins were eluted in buffer A supplemented with 2.5 mM *d*-desthiobiotin. StrepTactin XT Sepharose eluates were incubated with Ni-NTA agarose (Qiagen) for 1 h at 4 °C in buffer A with 20 mM imidazole before extensive washing in the same buffer and elution in buffer A with 250 mM imidazole. Depending on the downstream applications for the sample, tags were either left intact or cleaved off by overnight incubation at 4 °C with TEV protease (made in-house). Finally, samples were applied to a Superdex 200 Increase 10/300 GL column (GE Healthcare) in buffer A. Purified proteins were flash-frozen and stored at −80 °C.

For expression of the aPKCι^KD^, a recombinant baculovirus was generated for coexpression with phosphoinositide-dependent kinase 1 (PDK1), a priming kinase for aPKCι. Sequences for aPKCι residues 248–596, including an N-terminal GST tag with 3C protease cleavage site and untagged PDK1, were amplified from previous constructs of ours and inserted into the MultiBac pFL vector (www.addgene.org). Baculoviruses generated in *Sf*21 cells using standard protocols were used to infect *Sf*21 cells at a multiplicity of infection of 2. Cells were harvested by centrifugation after 3 days of shaking at 125 rpm at 27 °C and fully primed aPKCι^KD^ was purified as described previously^5^.

### aPKCι–Par6α–Llgl1 cryo-EM grid preparation and data collection

First, 4 µl of aPKCι–Par6α–Llgl1 complex at a concentration of 0.4 mg ml^−1^ was incubated with AMP-PNP and applied to R1.2/1.3 Quantifoil 300-mesh copper grids that were glow-discharged for 45 s at 45 mA. Grids were blotted for 2.5 s at 100% humidity using an FEI Vitrobot MK IV. Data were collected on a Titan Krios transmission EM instrument operated at 300 keV. Data were collected using a Gatan K2 summit direct electron detector operating in counting mode with a GIF quantum energy filter operating in zero-loss mode. Videos were collected with 8-s exposures dose-fractionated into 40 frames with a total dose of 48.1 e^−^ per Å^2^ and a calibrated pixel size of 0.82 Å. A total of 3,407 videos were collected with a defocus range of −3.5 µM to −1.5 µM.

### aPKCι–Par6α–Llgl1 cryo-EM image processing

MotionCor2 and ctffind 4.1 were used for motion correction and contrast transfer function estimation, respectively^39,40^. A total of 3,407 micrographs were selected for further processing. Semiautomated picking with Xmipp3 and particle extraction in RELION-3 yielded 47,516 particles from 1,000 micrographs^41^. After reference-free 2D classification in RELION-3, eight 2D classes were selected and used as templates for reference-based particle picking in Gautomatch (https://github.com/JackZhang-Lab/EM-scripts). A total of 1,069,057 particles were extracted with twofold binning and submitted to eight rounds of 2D classification in RELION-3. After one round of 2D classification in cryoSPARCv2, a subset of 48,000 particles was used for ab initio 3D model generation in cryoSPARCv2 (ref. ^42^). Three models were selected and used as references for 3D classification in RELION-3, this approach yielded 121,194 particles in a single stable 3D class. Particles were re-extracted with the original unbinned pixel size of 0.82 Å in a 280 × 280-pixel box before 3D autorefinement and Bayesian polishing in RELION-3. The polished particles were refined to 3.67-Å resolution using nonuniform refinement in cryoSPARCv2. Finally, the half-maps from cryoSPARCv2 refinement were used as inputs for density modification using phenix.resolve-cryoem^43^.

### aPKCι–Par6α–Llgl1 model building

Homology models of each individual Llgl1 β-propeller and the human Par6α PDZ domain were generated using Modeller^44^ using existing crystal structures of Llgl2 (PDB 6N8Q) and the mouse Par6^PDZ^ domain (PDB 1NF3), respectively^13,45^. These models, along with the aPKCι^KD^ crystal structure reported here, were rigid-body docked into the cryo-EM density using UCSF Chimera^46^. The resulting composite model was subjected to real-space refinement in PHENIX using the input models as reference model restraints before manual rebuilding in Coot^43,47^. The Llgl1 loop (10–11) and linkers between the Llgl1 β-propellers were built de novo in Coot. Further manual rebuilding in Coot and real-space refinement in PHENIX yielded a model comprising residues 15–951 for Llgl1, residues 154–252 for Par6α and residues 248–585 for aPKCι.

### aPKCι–Llgl2 substrate peptide crystallization and structure solution

aPKCι^KD^ was concentrated to 4 mg ml^−1^ and incubated with 1 mM MgCl_2_ and a threefold molar excess of both AMP-PCP and a peptide including residues 644–672 of Llgl2. Crystals grew at 27 °C in 25% (v/v) MPD, 25% (v/v) PEG 1000, 25% (v/v) PEG3350, 0.3 M NaNO_3_, 0.3 M Na_2_HPO_4_, 0.3 M (NH_4_)_2_SO_4_ and 0.1 M MES–imidazole pH 6.5. Native data were collected on beamline I04 at the Diamond Light Source. Data were scaled using DIALS^48^ and phases were estimated by molecular replacement using Phaser^49^ with PDB 3A8W as a search model. The crystals had two copies of aPKCι–Llgl2 peptide in the asymmetric unit. The Llgl2 substrate peptide was manually built using Coot and the structure was refined at 3.15 Å to an *R*_work_ of 0.218 and an *R*_free_ of 0.287 with tight geometry using Coot and PHENIX^43,47^. The final model included Llgl2 residues 657–666, AMP-PNP and human aPKCι^KD^ residues 240–578.

### Immunoprecipitation and pulldown assays

HEK293T or DLD1-FlpIn-TREx cells expressing GFP-tagged Llgl2 WT or mutants were lysed in 50 mM Tris pH 7.4, 150 mM NaCl, 1% Triton and 0.5 mM TCEP supplemented with phosphatase inhibitors (PhosStop) and protease inhibitors (cOmplete, Roche) and incubated with GFP-Trap magnetic agarose (Chromotek) for 2 h at 4 °C. Beads were washed once in lysis buffer containing 260 mM NaCl and twice in TBS. Proteins were eluted in 1× SDS NuPAGE loading buffer (Thermo Fisher Scientific). Electrophoresis and western blotting (wet transfer) were performed according to standard protocols and imaging was performed using an LAS-4000 charge-coupled device camera (GE healthcare).

### In vitro dissociation assays

To monitor dissociation of Myc-tagged aPKCι and FLAG-tagged Par6α from GFP–Llgl2, the three proteins were coexpressed in FreeStyle 293-F cells and GFP-Trap was performed as described above using GFP-Trap magnetic agarose (Chromotek). The immunoprecipitated complex was then added to reaction buffer (20 mM Tris pH 7.4, 150 mM NaCl and 10 mM MgCl_2_,) supplemented with one or more of the following components: Cdc42 (Q61L mutant; 1.4 μM), GTPγS (100 μM), Crb3 PBM peptide (Biotin-Ahx-LPPEERLI-COOH; 100 μM), ADP (10 μM) and ATP (100 μM). Reactions were performed at 30 °C for endpoint measurements and at 16 °C for kinetic measurements. The supernatant was separated from the immobile fraction using a magnet and the magnetic beads were washed once with TBS supplemented with 0.1% Tween-20 before the addition of 2× NuPAGE loading buffer (Thermo Fisher Scientific).

### Immunofluorescence staining and confocal microscopy

DLD1-FlpIn-TREx cells stably harboring genes for GFP-tagged WT and mutant forms of Llgl2 were seeded on 13-mm glass coverslips at a density of 0.1 × 10^6^ cells. Then, 24 h after plating, cultures were induced with 200 ng ml^−1^ doxycycline for 16 h or left uninduced. Cells were fixed with 4% PFA and permeabilized with PBS + 0.1% Triton X-100. Coverslips were then blocked in 3% BSA in PBS and incubated for 2 h with anti-TJP1 antibody (1:500) or anti-TGN46 antibody (1:500), washed three times in PBS and subsequently incubated for 2 h at room temperature with goat anti-rabbit 555 (1:1,500) or donkey anti-sheep 647 (1:1,500) antibodies, respectively (Thermo Fisher Scientific). Coverslips were mounted using Prolong gold with DAPI (Thermo Fisher Scientific) and imaged. All images were acquired using an inverted laser scanning confocal microscope (Carl Zeiss LSM 880 operated using Zen Black software) using a ×63 or ×40 Plan-APOCHROMAT DIC oil-immersion objective. Images shown in figures were processed in ZEN Blue edition (Zeiss). All images were batch-processed to adjust brightness and contrast. Scoring of ZO1 staining was performed manually by counting cells that were fully enclosed by ZO1 staining (intact ZO1), displayed partial or fragmented enclosure (discontinuous ZO1) or lacked ZO1 enclosure (loss of ZO1).

### *Drosophila* stocks and genetics

*Drosophila melanogaster* flies were grown using cornmeal, agar, molasses and yeast medium in incubators at temperatures of 18 °C and 25 °C with controlled photoperiod and humidity. The *lgl*^*27S3*^-null mutant allele (Bloomington *Drosophila* Stock Center (BDSC), 41561) and the following UAS transgenic lines were used: UAS-Lgl-GFP^32^, UAS-Lgl^ASA^-GFP (this paper), UAS-Lgl^RPYSR^-GFP (this paper) and UAS-Lgl^NE^-GFP (this paper). *GR1-Gal4* was used to induce expression of UAS transgenes in the follicular epithelium. The GR1-Gal4 driver shows mild expression during during stages 4–7 of oogenesis. All experiments were carried out at 25 °C to promote mild expression mediated by *GR1-Gal4*. The Flp-*FRT*-mediated mitotic recombination system was used for clonal analyses in the follicle epithelium. Mosaic clones were induced by heat shock at 37 °C in flies with the following genotypes:

**Table:** 

Fig. 4g	$\frac{\rm{hsFlp}}{+};\frac{{{lgl}}^{27S3}\;{FRT}40\;{\rm{{UAS}-{Lgl}-{GFP}}}}{{{\rm{nlsRFP}}\; FRT}40};\frac{{GR}1-{Gal}4}{+}$
Fig. 4h	$\frac{\rm{hsFlp}}+;\frac{{{lgl}}^{27S3}\;{FRT}40\;{{{\rm{UAS}}-{\rm{Lgl}}^{\rm{NE}}-{\rm{GFP}}}}}{{{\rm{nlsRFP}}\; FRT}40};\frac{{GR}1-{Gal}4}{+}$
Fig. 4i	$\frac{\rm{hsFlp}}{+};\frac{{{lgl}}^{27S3}\;{FRT}40\;{\rm{UAS}-{{Lgl}}^{{RPYSR}}-{GFP}}}{{{\rm{{nlsRFP}}}\; FRT}40};\frac{{GR}1-{Gal}4}{+}$
Fig. 4j	$\frac{\rm{hsFLP}}{+};\frac{{{lgl}}^{27S3}\;{FRT}40\;{\rm{UAS}-{{Lgl}}^{{ASA}}-{GFP}}}{{{\rm{{nlsRFP}}}\; FRT}40};\frac{{GR}1-{Gal}4}{+}$

### Cloning and transgenesis of Lgl mutants

Site-directed mutagenesis was performed using Champalimaud Foundation’s Molecular and Transgenic Tools Platform (MTTP) to introduce D502R and D506R substitutions (GATCCTTATTCAGAT to CGTCCTTATTCACGT) in Lgl^RPYSR^, the I695N substitution (ATA to AAC) in Lgl^NE^ and L656A and R653A substitutions (CTGTCTCGT to GCGTCTGCT) in Lgl^ASA^ using pENTR-Lgl as a template^25^. The GFP-tagged constructs were obtained using LR clonase II to mediate the recombination into pUASt.attb.WG and then inserted into the attP-VK18 landing site on chromosome II (BDSC, 9736) through PhiC31 site-specific transgenesis (BestGene). This enabled comparable expression levels of the different GFP-tagged mutant versions as all were inserted in the same genomic locus that was used for the control version.

### Fixation and immunofluorescence of *Drosophila* egg chambers

Ovaries of well-fed *Drosophila* females were fixed in 4% PFA (in PBS) for 20 min, washed three times for 10 min in PBT (PBS with 0.05% Tween-20), blocked with PBT-10 (PBT supplemented with 10% BSA) and then incubated overnight with primary antibodies in PBT-1 (PBT supplemented with 1% BSA). After four 30-min washes in PBT-1, ovaries were incubated with secondary antibodies in PBT-0.1 (PBT supplemented with 0.1% BSA) for 150 min, washed three times for 10 min with PBT and mounted in Vectashield with DAPI (Vector Laboratories). Rabbit anti-aPKCζ (1:500; c-20, Santa Cruz Biotechnology) was used as the primary antibody.

### Image acquisition and analysis in the *Drosophila* follicular epithelium

Immunostainings were analyzed using a confocal microscope Leica TCS SP8 (Leica Microsystems) with a PL APO ×63/1.30 glycerol objective and the LAS X software. Image processing and quantifications were performed using Fiji^50^. Data processing was performed in Excel while statistical analysis and graphical representations were performed using GraphPad Prism 8 tools. To analyze the ability of different Lgl mutants to recapitulate Lgl function on epithelial apical–basal organization, we monitored whether the expression of Lgl transgenes carrying different mutations would rescue the fully penetrant multilayering phenotype of tissue homozygous for the *lgl*^*27S3*^-null allele^32^. Multilayering (defined as three or more epithelial cells piling on top of the epithelial layer) was scored by inspecting midsagittal cross-sections of stage 4–7 egg chambers. Only egg chambers with large mutant clones (occupying more than one quarter of the whole egg chamber mutant) were considered in the analysis. The developmental stage of egg chambers was determined by measuring their area in midsagittal cross-sections as a proxy for size. To define the area intervals corresponding to each developmental stage, we staged control egg chambers stained for aPKC and overexpressing UAS-Lgl-GFP flies according to phenotypic characteristics and correlated area size with the developmental stage. For each independent experiment, the analyzed egg chambers were obtained from a minimum of ten flies per genotype.

### Reporting summary

Further information on research design is available in the Nature Portfolio Reporting Summary linked to this article.

## Online content

Any methods, additional references, Nature Portfolio reporting summaries, source data, extended data, supplementary information, acknowledgements, peer review information; details of author contributions and competing interests; and statements of data and code availability are available at 10.1038/s41594-024-01425-0.

## Supplementary information

Supplementary Information Supplementary Table 1.

Reporting Summary

## Source data

Source Data Fig. 2 Unprocessed western blots.

Source Data Fig. 2 Statistical source data.

Source Data Fig. 3 Unprocessed western blots.

Source Data Fig. 3 Statistical source data.

Source Data Fig. 4 Unprocessed western blots.

Source Data Fig. 4 Statistical source data.

Source Data Fig. 5 Unprocessed western blots.

Source Data Fig. 5 Statistical source data.

Source Data Extended Data Fig. 2 Unprocessed western blots.

Source Data Extended Data Fig. 3 Unprocessed western blots.

Source Data Extended Data Fig. 3 Statistical source data.

Source Data Extended Data Fig. 4 Unprocessed western blots.

Source Data Extended Data Fig. 4 Statistical source data.

## Data Availability

The cryo-EM map of aPKCι–Par6α–Llgl1 complex is available from the EM Data Bank (accession number EMD-18877). The structure coordinate file for the fitted aPKCι–Par6α–Llgl1 model is available from the PDB database (accession number 8R3Y). The structure coordinate file for the fitted aPKCι^KD^ bound to Llgl2 P-site peptide is available from the PDB database (accession number 8R3X). All biological materials generated in this manuscript are available from the authors upon request Source data are provided with this paper.
