# Supplementary material for: Capture, mutual inhibition and release mechanism for aPKC–Par6 and its multisite polarity substrate Lgl
Source: Nat Struct Mol Biol. 2025 Jan 6;32(4):729–39. doi: 10.1038/s41594-024-01425-0 (PMC11996676; doi:10.1038/s41594-024-01425-0)
Supplement: Supplementary file 1 — Supplementary Table 1. [file 41594_2024_1425_MOESM1_ESM.pdf]

nature structural & molecular biology

Supplementary information https://doi.org/10.1038/s41594-024-01425-0

Capture, mutual inhibition and release mechanism for aPKC–Par6 and its multisite polarity substrate Lgl

In the format provided by the authors and unedited

Contents

Supplementary information table 1. 3

Plasmids used in this study:	
plasmid name:	source:
pEGFP-C1-LLGL2	Mark Linch/Tony Pawson (PMID: 24045153)
pcDNA3.1-2xStrep-TEV-LLGL1	this study
pCDNA3.1-His_TEV-PARD6A	this study
pcDNA3.1-FLAG-PARD6A	this study
pcDNA3.1-PRKCI	this study
pcDNA3.1-Myc-PRKCI	Cobbaut et al. (PMID: 37211093)
pcDNA5-TO-GFP-LLGL2	this study
**Mutagenesis primers used in this study:**	
primer name:	sequence:
LLGL2 - AAS_FW [645/649]	GGCCCTCAAGAAGGCCTTGCGTCAGTCATTCCGCC
LLGL2 - AAS_RV [645/649]	GCCTTCTTGAGGGCCTTGACGCGGGAGAGTGG
LLGL2 - S3A_FW [645/649/653]	CAAGAAGGCCTTGCGTCAGGCATTCCGCCGGATGCGTCGG
LLGL2 - S3A_RV [645/649/653]	CGCAAGGCCTTCTTGAGGGCCTTGACGCGGGAGAGTGGG
LLGL2 - SSA_FW [653]	CAAGAAGTCCTTGCGTCAGGCATTCCGCCGGATGCGTCGG
LLGL2 - SSA_RV [653]	CGCAAGGACTTCTTGAGGGACTTGACGCGGGAGAGTGGG
LLGL2 - SAA_FW [649/653]	CAAGAAGGCCTTGCGTCAGGCATTCCGCCGGATGCGTCGG
LLGL2 - SAA_RV [649/653]	CGCAAGGCCTTCTTGAGGGACTTGACGCGGGAGAGTGGG
LLGL2 - ASA_FW [645/653]	CAAGAAGTCCTTGCGTCAGGCATTCCGCCGGATGCGTCGG
LLGL2 - ASA_RV [645/653]	CGCAAGGACTTCTTGAGGGCCTTGACGCGGGAGAGTGGG
LLGL2_R698A_FW	TGTGCAGGCCAAGATCGAGGCTCGCTCGG
LLGL2_R698A_RV	ATCTTGGCCTGCACAGGCGCCAGCTC
LLGL2_I700N_FW	GCGCAAGAACGAGGCTCGCTCGGCAGAGG
LLGL2_I700N_RV	GCCTCGTTCTTGCGCTGCACAGGCGC
LLGL2_E701Q_FW	CAAGATCCAGGCTCGCTCGGCAGAGGAC
LLGL2_E701Q_RV	CGAGCCTGGATCTTGCGCTGCACAGGC
LLGL2_E701A_FW	CAAGATCGCGGCTCGCTCGGCAGAGGAC
LLGL2_E701A_RV	CGAGCCGCGATCTTGCGCTGCACAGGC
LLGL2_RKIE.AKNQ_FW	AGGCCAAGAACCAGGCTCGCTCGGCAGAGGAC

LLGL2_RKIE.AKNQ_RV	CCTGGTTCTTGGCCTGCACAGGCGCCAGCTC
LLGL2_LxR_AxA_FW	CCCAGCCTCCGCCGTCAAGTCCCTCAAGAAGTCCTTGC
LLGL2_LxR_AxA_RV	ACGGCGGAGGCTGGGCCCTCCAAGGCCAGC
LLGL2_RPYSR_FW	TAGGCCCTACAGTAGGGACCCCCGGCTGGGCATCC
LLGL2_RPYSR_RV	CTACTGTAGGGCCTAAAGGAGCCCACCTTGCGG
PKCi_DIPD_FW	TTGACATACCAGACTCTCTGTCTGTAAAAGCTGC
PKCi_DIPD_RV	AGTCTGGTATGTCAATTTGTTTTTCCAAAATAACTTGG
dLGL 651-653 ASA FW	ATCTTACTGGAGCAGGAGAGCAGGCGTCTGCTCGAAAGTCTTTTAAGAAATCAT
dLGL 651-653 ASA RV	ATGATTTCTTAAAAGACTTTCGAGCAGACGCCTGCTCTCCTGCTCCAGTAAGAT
dLGL D502R/D506R FW	CCGCCATTTCGGAAATCAGGACTTTTTCGTCCTTATTCACGTGACCCTCGTTTAG
dLGL D502R/D506R RV	CTAAACGAGGGTCACGTGAATAAGGACGAAAAAGTCCTGATTTCCGAAATGGCGG
dLGL I695N FW	GACCCGTCGAGAGGCAAAACGAGGCTCGTTGTGC
dLGL I695N RV	GCACAACGAGCCTCGTTTTGCCTCTCGACGGGTC

Supplementary information table 1.

Plasmids and primers used in this study.
