# Supplementary material for: Capture, mutual inhibition and release mechanism for aPKC–Par6 and its multisite polarity substrate Lgl
Source: Nat Struct Mol Biol. 2025 Jan 6;32(4):729–39. doi: 10.1038/s41594-024-01425-0 (PMC11996676; doi:10.1038/s41594-024-01425-0)
Supplement: Supplementary file 2 — Reporting Summary [file 41594_2024_1425_MOESM2_ESM.pdf]

nature portfolio

Corresponding author(s): Mathias Cobbaut, Neil McDonald

Last updated by author(s): Aug 15, 2024

Reporting Summary

Nature Portfolio wishes to improve the reproducibility of the work that we publish. This form provides structure for consistency and transparency in reporting. For further information on Nature Portfolio policies, see our Editorial Policies and the Editorial Policy Checklist.

Statistics

For all statistical analyses, confirm that the following items are present in the figure legend, table legend, main text, or Methods section.

n/a	Confirmed	
☐	☒	The exact sample size (*n*) for each experimental group/condition, given as a discrete number and unit of measurement
☐	☒	A statement on whether measurements were taken from distinct samples or whether the same sample was measured repeatedly
☐	☒	The statistical test(s) used AND whether they are one- or two-sided<br>*Only common tests should be described solely by name; describe more complex techniques in the Methods section.*
☒	☐	A description of all covariates tested
☐	☒	A description of any assumptions or corrections, such as tests of normality and adjustment for multiple comparisons
☐	☒	A full description of the statistical parameters including central tendency (e.g. means) or other basic estimates (e.g. regression coefficient) AND variation (e.g. standard deviation) or associated estimates of uncertainty (e.g. confidence intervals)
☐	☒	For null hypothesis testing, the test statistic (e.g. *F*, *t*, *r*) with confidence intervals, effect sizes, degrees of freedom and *P* value noted<br>*Give P values as exact values whenever suitable.*
☒	☐	For Bayesian analysis, information on the choice of priors and Markov chain Monte Carlo settings
☒	☐	For hierarchical and complex designs, identification of the appropriate level for tests and full reporting of outcomes
☒	☐	Estimates of effect sizes (e.g. Cohen's *d*, Pearson's *r*), indicating how they were calculated

Our web collection on statistics for biologists contains articles on many of the points above.

Software and code

Policy information about availability of computer code

Data collection	Zen Black (2.3 SP1), LAS X, ImageQuant Las 4000 (v1.3)
Data analysis	Excel v2108, GraphPad Prism versions 8 and 10, Xmipp v3.0, Relion v3.1, Scipion v3.0, CryoSPARC-2, CTFFIND v4.1 DIALS/DUI, Phaser 2.8.3, Modeller 9.24, Phenix.Refine 1.20.1 , Coot v0.9.8, MolProbity USCF ChimeraX 1.6, USCF Chimera 1.16, Pymol, ImageJ 1.54f, FIJI, ZEN Blue Zeiss 3.4.91

For manuscripts utilizing custom algorithms or software that are central to the research but not yet described in published literature, software must be made available to editors and reviewers. We strongly encourage code deposition in a community repository (e.g. GitHub). See the Nature Portfolio guidelines for submitting code & software for further information.

Data

Policy information about availability of data

All manuscripts must include a data availability statement. This statement should provide the following information, where applicable:

- Accession codes, unique identifiers, or web links for publicly available datasets
- A description of any restrictions on data availability
- For clinical datasets or third party data, please ensure that the statement adheres to our policy

we have provided the following data availability statement in the manuscript:

The cryo-EM map of aPKC-Par6-Llgl1 complex is available in the Electron Microscopy Data Bank (accession number EMD-18877). The structure coordinate file for the fitted aPKC-Par6-Llgl1 model is available in the Protein Data Bank database (accession number 8R3Y). The structure coordinate file for the fitted aPKC kinase domain bound to Llgl2 P-site peptide is available in the PDB database (accession number 8R3X). All biological materials generated in this manuscript are available from the authors upon request. Further information on the research design is available in the Nature Research Reporting Summary linked to this article. Source data are provided with this paper.

Research involving human participants, their data, or biological material

Policy information about studies with human participants or human data. See also policy information about sex, gender (identity/presentation), and sexual orientation and race, ethnicity and racism.

Reporting on sex and gender	N/A
Reporting on race, ethnicity, or other socially relevant groupings	N/A
Population characteristics	N/A
Recruitment	N/A
Ethics oversight	N/A

Note that full information on the approval of the study protocol must also be provided in the manuscript.

Field-specific reporting

Please select the one below that is the best fit for your research. If you are not sure, read the appropriate sections before making your selection.

☒ Life sciences ☐ Behavioural & social sciences ☐ Ecological, evolutionary & environmental sciences

For a reference copy of the document with all sections, see nature.com/documents/nr-reporting-summary-flat.pdf

Life sciences study design

All studies must disclose on these points even when the disclosure is negative.

Sample size	No statistics were employed to predetermine the sample size. The biochemical and cell-based as well as the in vivo samples sizes were chosen as such to ensure reproducibility of the observations. For Cryo-EM 121,194 particles were selected for the final EM reconstruction, as this amount resulted in the reported resolution and map quality.
Data exclusions	No data was excluded from the in vitro, cell-based and in vivo analyses. For cryo-EM experiments, consistent with standard protocols, picked particles that contributed to 2D classes and 3D reconstructions with lower resolution were removed.
Replication	In-vitro, cell-based and in vivo assays were successfully repeated n times as indicated in the figure legends or material and methods section.
Randomization	Allocation of experimental conditions (e.g. cellular treatments) were random. The other assays used in this study are not subject to the systematic variation which demands randomisation, or were impossible to randomize because of the practical nature of these experiments..
Blinding	For microscopy acquisition conditions were coded with a shorthand code corresponding to a cellular condition recorded in a separate file. For other data acquisition (e.g. biochemical experiments) blinding was not possible or relevant because of the practical nature of these experiments. For Drosophila experiments, we used clonal mutant analysis and had fluorescent labels in the proteins of interest, so that we could distinguish control and mutant cells in tissues that were mosaic for different cell populations.

Reporting for specific materials, systems and methods

We require information from authors about some types of materials, experimental systems and methods used in many studies. Here, indicate whether each material, system or method listed is relevant to your study. If you are not sure if a list item applies to your research, read the appropriate section before selecting a response.

Materials & experimental systems

n/a	Involved in the study
☐	☒ Antibodies
☐	☒ Eukaryotic cell lines
☒	☐ Palaeontology and archaeology
☐	☒ Animals and other organisms
☒	☐ Clinical data
☒	☐ Dual use research of concern
☒	☐ Plants

Methods

n/a	Involved in the study
☒	☐ ChIP-seq
☒	☐ Flow cytometry
☒	☐ MRI-based neuroimaging

Antibodies

Antibodies used

Anti-Myc (9B11) Antibody 1/1500 Cell Signalling Technologies #2276
Anti-GFP (4B10) Antibody 1/3000 Cell Signalling Technologies #2955
Anti-His (Rabbit) Antibody 1/1000 Cell Signalling Technologies #2365
Phospho-Llgl1/2 S663 Antibody 1/2000 Cell Signalling Technologies commissioned
Anti-FLAG M2 Antibody 1/2000 Sigma F3165
Phospho-LLGL1/2 S650/654 Antibody 1/1500 Abgent AP2198a
Anti-LLGL1 mAb (5G2) Antibody 1/2000 Abnova 00003996-MO1
Anti-LLG2 Antibody 1/2000 Abcam ab73304
Par6B (B-10) Antibody 1/300 Santa Cruz sc-166405
Anti-TJP1 (ZO1) Antibody 1/500 Atlas Antibodies HPA001636
TGN46 antibody 1/500 BioRad AHP500GT
Rabbit Anti-aPKCzeta Antibody 1/500 Santa Cruz Biotechnology sc-17781

Secondary HRP-Linked Goat anti-Rabbit Cell Signalling Technologies #7074
Secondary HRP-Linked Horse anti-Mouse Cell Signalling Technologies #7076
Goat Anti-Rabbit 555 Antibody ThermoFisher Scientific A21428
Donkey anti-Sheep 647 Antibody ThermoFisher Scientific A21448

Validation

Antibodies were verified for the indicated species and applications by the respective manufacturer.
the validation statements can be found on the manufacturer's websites specified here:

Anti-Myc (9B11) https://www.cellsignal.com/products/primary-antibodies/myc-tag-9b11-mouse-mab/2276
Anti-GFP (4B10) https://www.cellsignal.com/products/primary-antibodies/gfp-4b10-mouse-mab/2955
Anti-His (Rabbit) https://www.cellsignal.com/products/primary-antibodies/his-tag-antibody/2365
Anti-FLAG M2 https://www.sigmaaldrich.com/GB/en/product/sigma/f3165
Phospho-LLGL1/2 S650/654 https://www.abcepta.com/products/AP2198a-Bi-Phospho-LLGL1-2-S655-659---S645-S649-Antibody
Anti-LLGL1 mAb (5G2) https://www.abnova.com/en-global/product/detail/H00003996-M01
Anti-LLG2 https://www.abcam.com/en-us/products/primary-antibodies/llgl2-antibody-ab73304
Par6B (B-10) https://www.scbt.com/p/pard6b-antibody-b-10
Anti-TJP1 https://www.atlasantibodies.com/products/primary-antibodies/triple-a-polyclonals/anti-tjp1-antibody-hpa001636/
TGN46 https://www.bio-rad-antibodies.com/polyclonal/human-tgn46-antibody-ahp500.html?f=purified
Rabbit Anti-aPKCzeta https://www.scbt.com/p/pkc-zeta-antibody-h-1

Llgl phospho-antibodies were additionally validated for specificity via western blot in this manuscript. We refer to Extended data Fig. 2c for the analysis of the specificity of these antibodies.

Eukaryotic cell lines

Policy information about cell lines and Sex and Gender in Research

Cell line source(s)

HEK293F (Thermo Fisher)
HEK293T (ATTC)
DLD1.FlpIN (Prof. Stephen Taylor, Manchester University)
Sf21 (Invitrogen)

Authentication

Carried out by the Cell Service Science Technology Platform at The Francis Crick Institute by STR profiling

Mycoplasma contamination

Mycoplasma testing of banked cell lines is carried out by Cell Service Science Technology Platform at The Francis Crick Institute with no reported positive results

Commonly misidentified lines
(See ICLAC register)

no commonly misidentified cell lines were used in the study

Animals and other research organisms

Policy information about studies involving animals; ARRIVE guidelines recommended for reporting animal research, and Sex and Gender in Research

Laboratory animals	The manuscript uses genetically modified strains of Drosophila melanogaster. Stage 4 to stage 7 egg chambers were dissected from 3 to 6 days old adult Drosophila melanogaster flies. Details of the experimental model are described in the methods.
Wild animals	N/A
Reporting on sex	N/A
Field-collected samples	N/A
Ethics oversight	N/A

Note that full information on the approval of the study protocol must also be provided in the manuscript.

Plants

Seed stocks	*Report on the source of all seed stocks or other plant material used. If applicable, state the seed stock centre and catalogue number. If plant specimens were collected from the field, describe the collection location, date and sampling procedures.*
Novel plant genotypes	*Describe the methods by which all novel plant genotypes were produced. This includes those generated by transgenic approaches, gene editing, chemical/radiation-based mutagenesis and hybridization. For transgenic lines, describe the transformation method, the number of independent lines analyzed and the generation upon which experiments were performed. For gene-edited lines, describe the editor used, the endogenous sequence targeted for editing, the targeting guide RNA sequence (if applicable) and how the editor was applied.*
Authentication	*Describe any authentication procedures for each seed stock used or novel genotype generated. Describe any experiments used to assess the effect of a mutation and, where applicable, how potential secondary effects (e.g. second site T-DNA insertions, mosiacism, off-target gene editing) were examined.*
