# Supplementary figures and images for: Capture, mutual inhibition and release mechanism for aPKC–Par6 and its multisite polarity substrate Lgl
Source: Nat Struct Mol Biol. 2025 Jan 6;32(4):729–39. doi: 10.1038/s41594-024-01425-0 (PMC11996676; doi:10.1038/s41594-024-01425-0)

Fig 2g

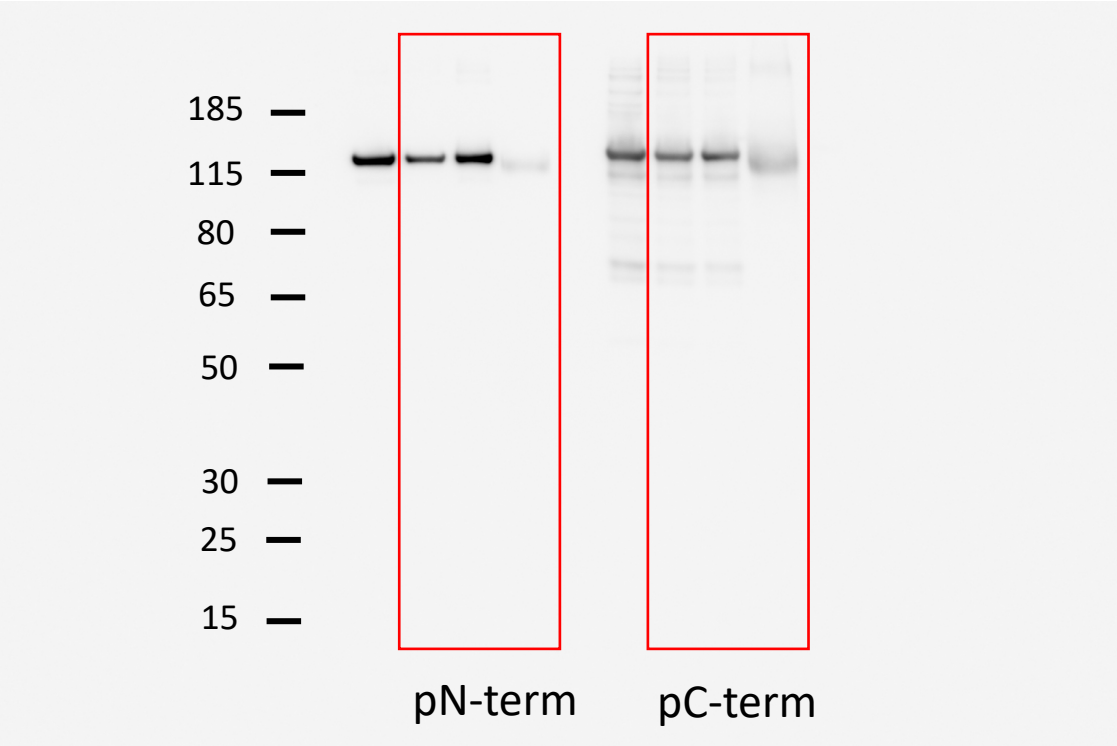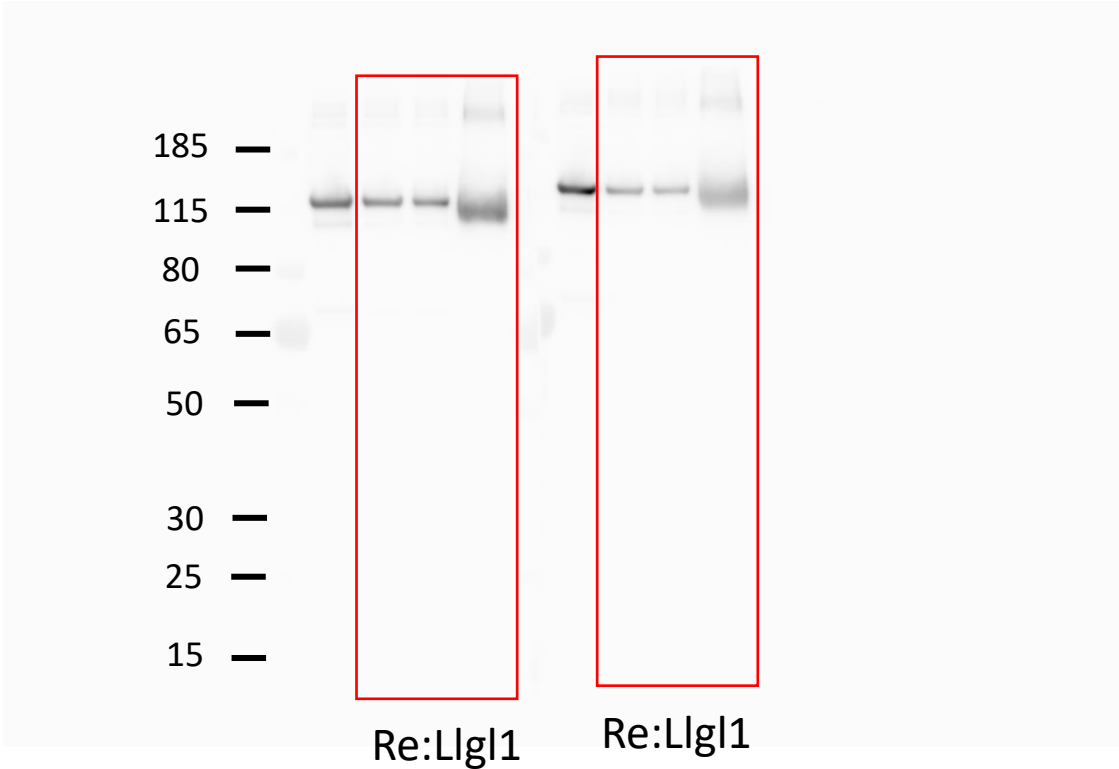

Supplement: Supplementary file 3 — Unprocessed western blots. [file 41594_2024_1425_MOESM3_ESM.pdf]

Fig 3b

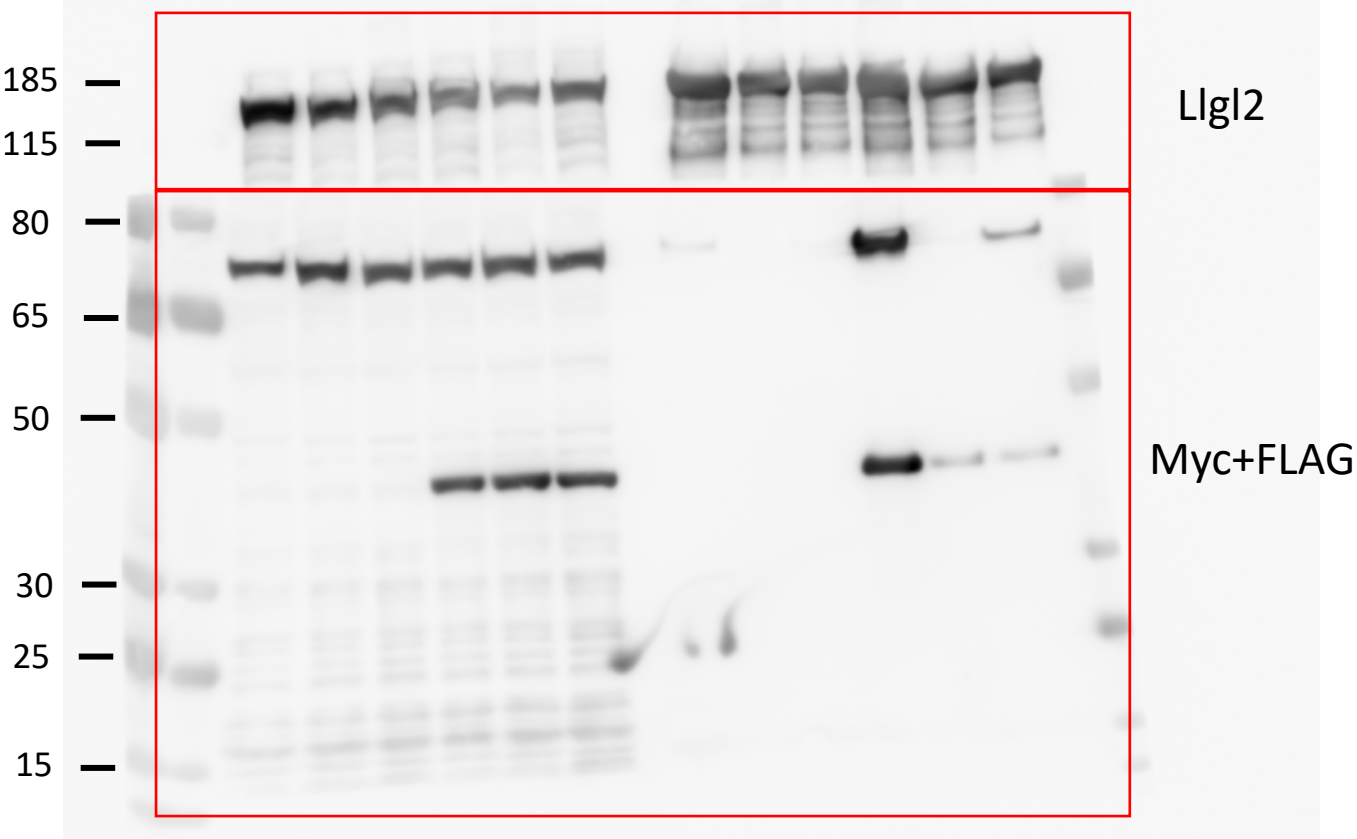

Fig 3c

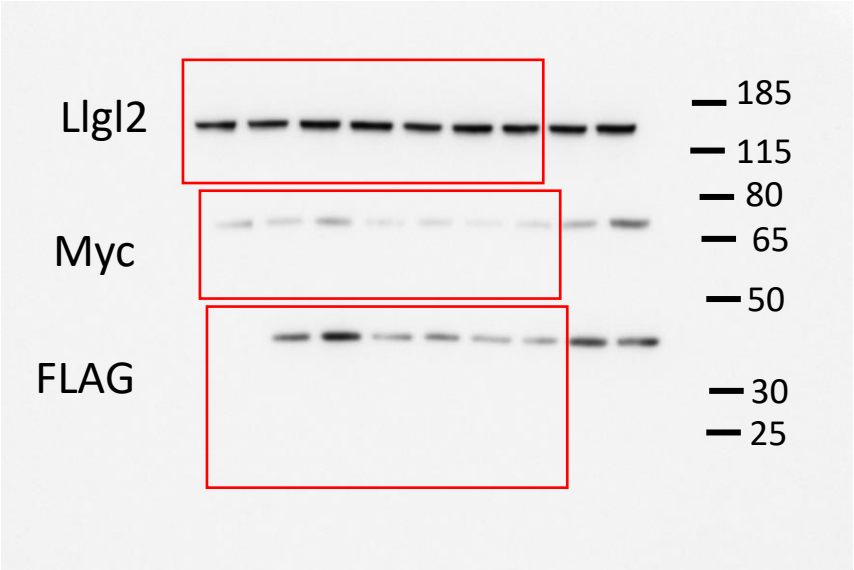

Lysates

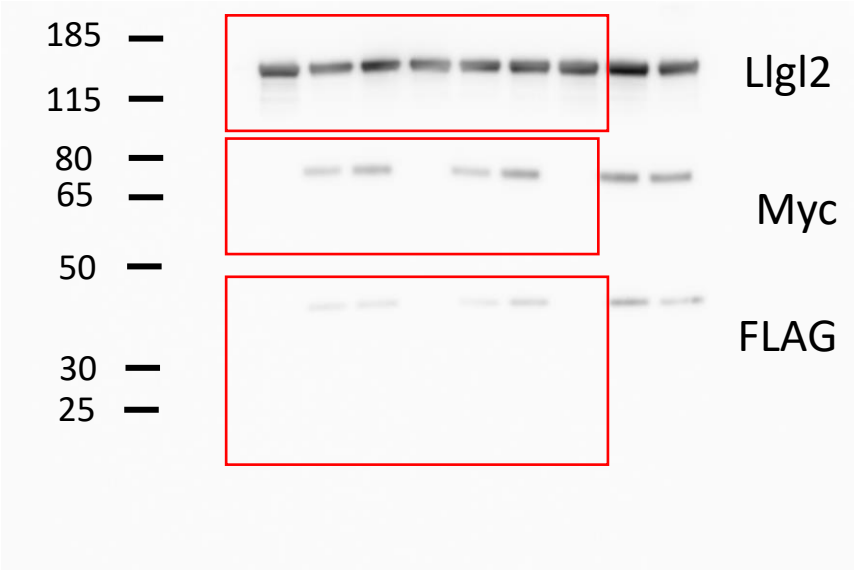

GFP-trap

Supplement: Supplementary file 5 — Unprocessed western blots. [file 41594_2024_1425_MOESM5_ESM.pdf]

Fig 4d

Lysates

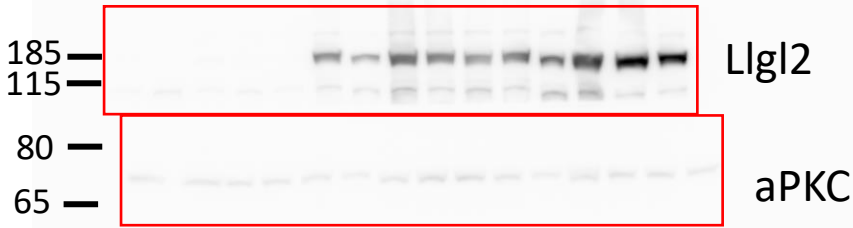

GFP-trap

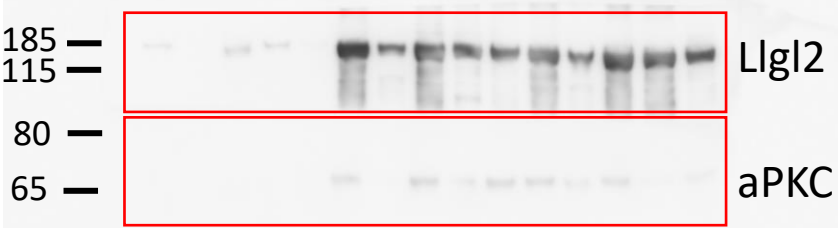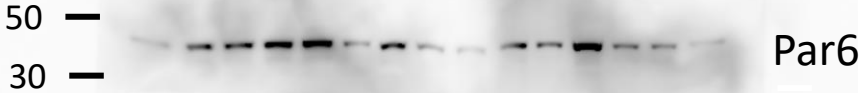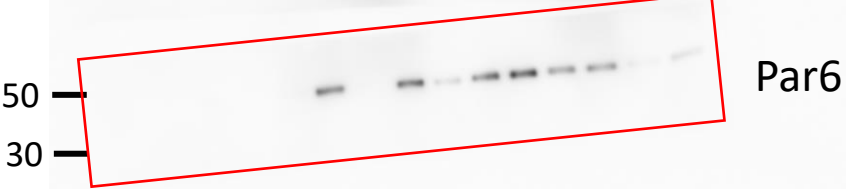

Fig 4e

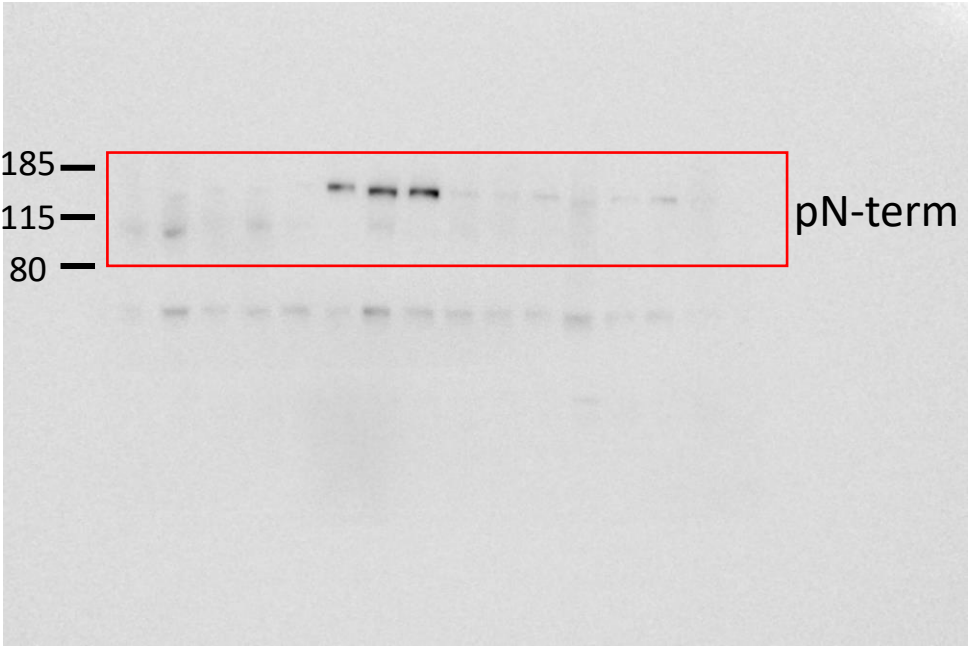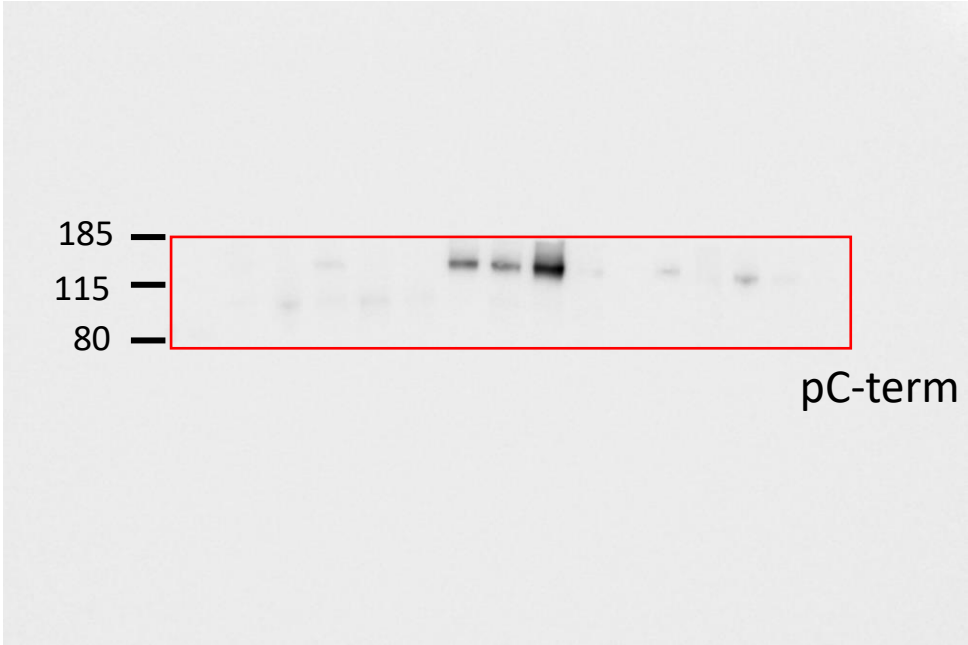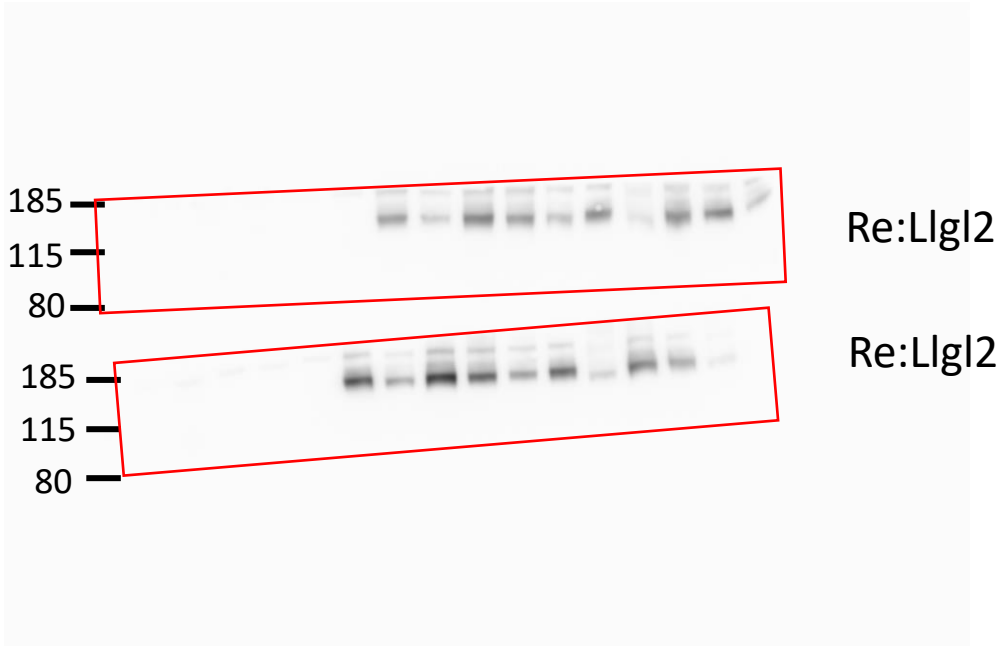

Supplement: Supplementary file 7 — Unprocessed western blots. [file 41594_2024_1425_MOESM7_ESM.pdf]

Fig 5b

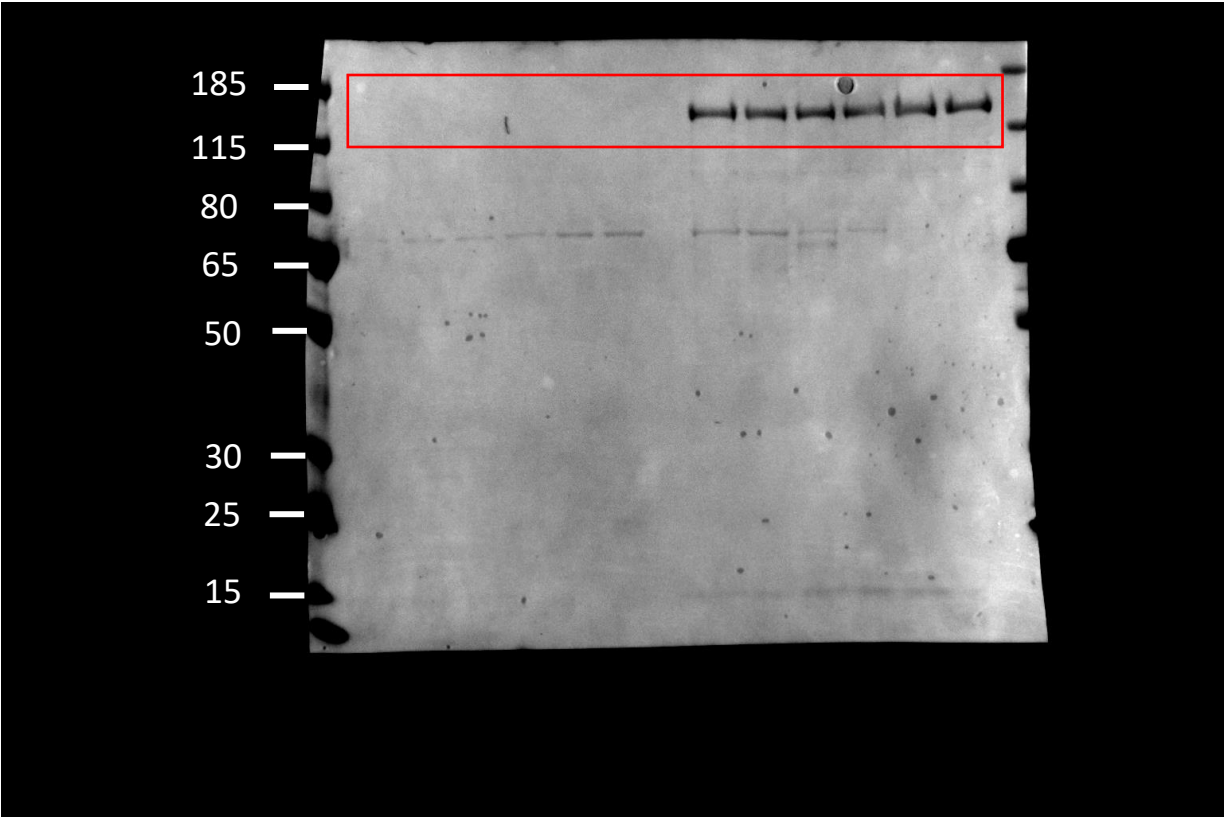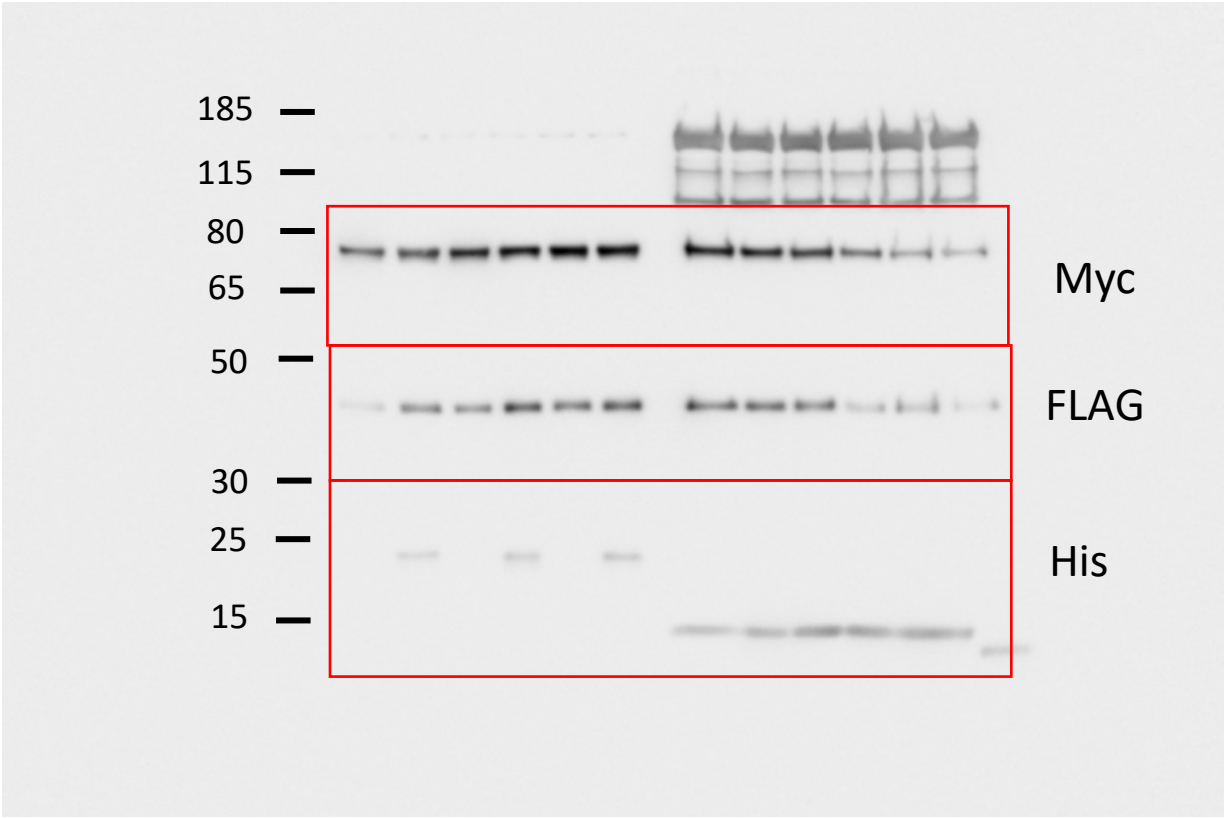

Fig 5c

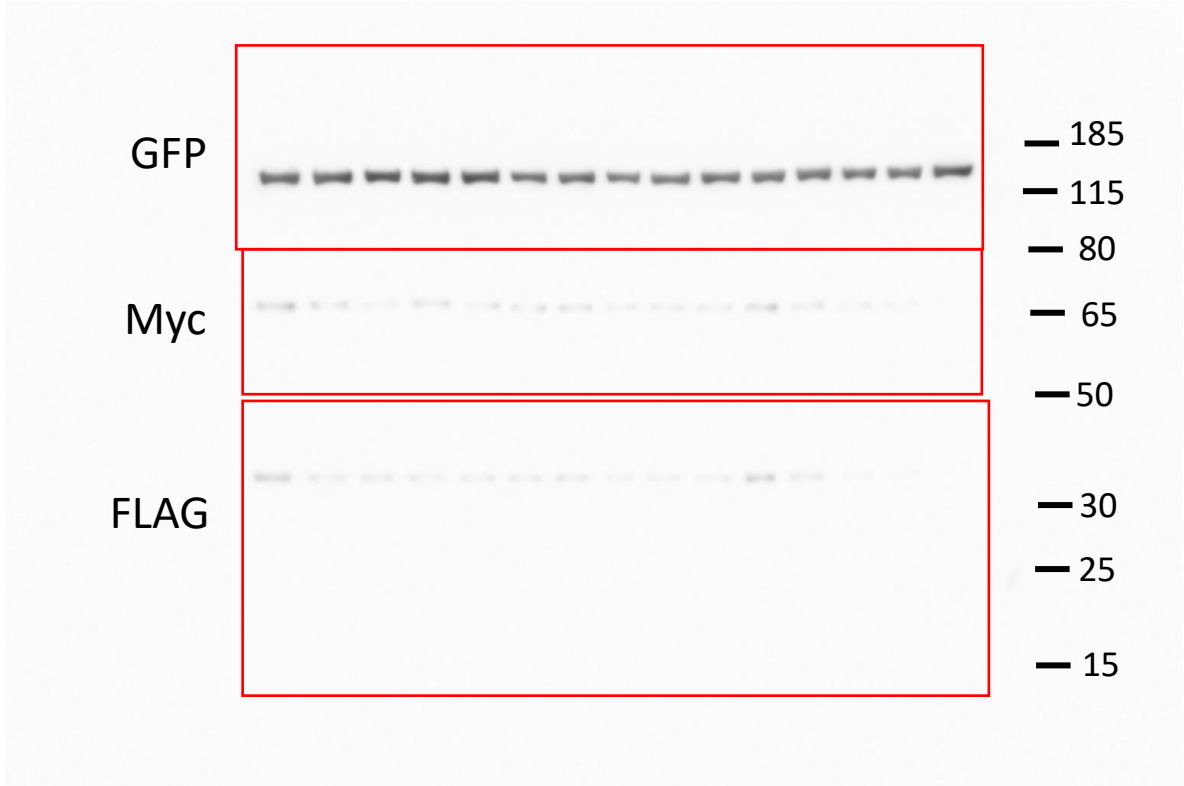

Supplement: Supplementary file 9 — Unprocessed western blots. [file 41594_2024_1425_MOESM9_ESM.pdf]

ED Fig 2c

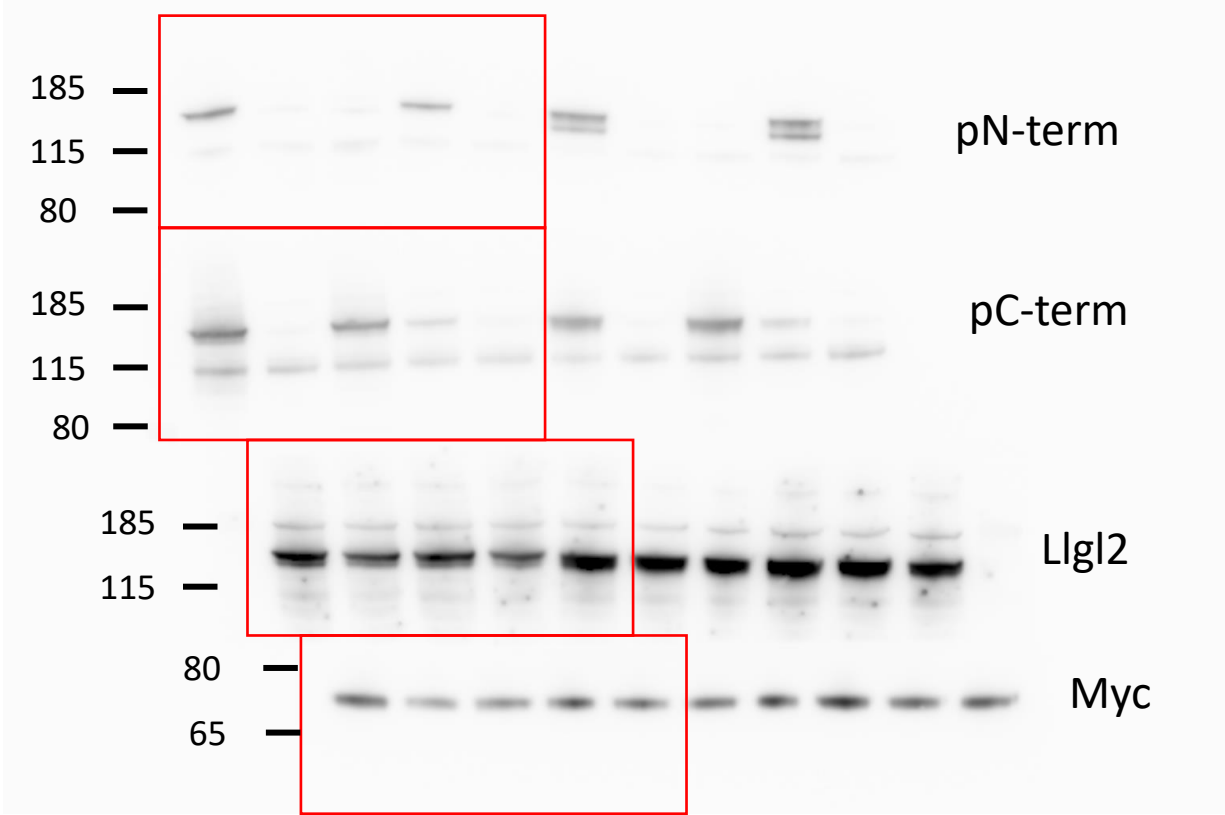

Supplement: Supplementary file 11 — Unprocessed western blots. [file 41594_2024_1425_MOESM11_ESM.pdf]

ED Fig 3d

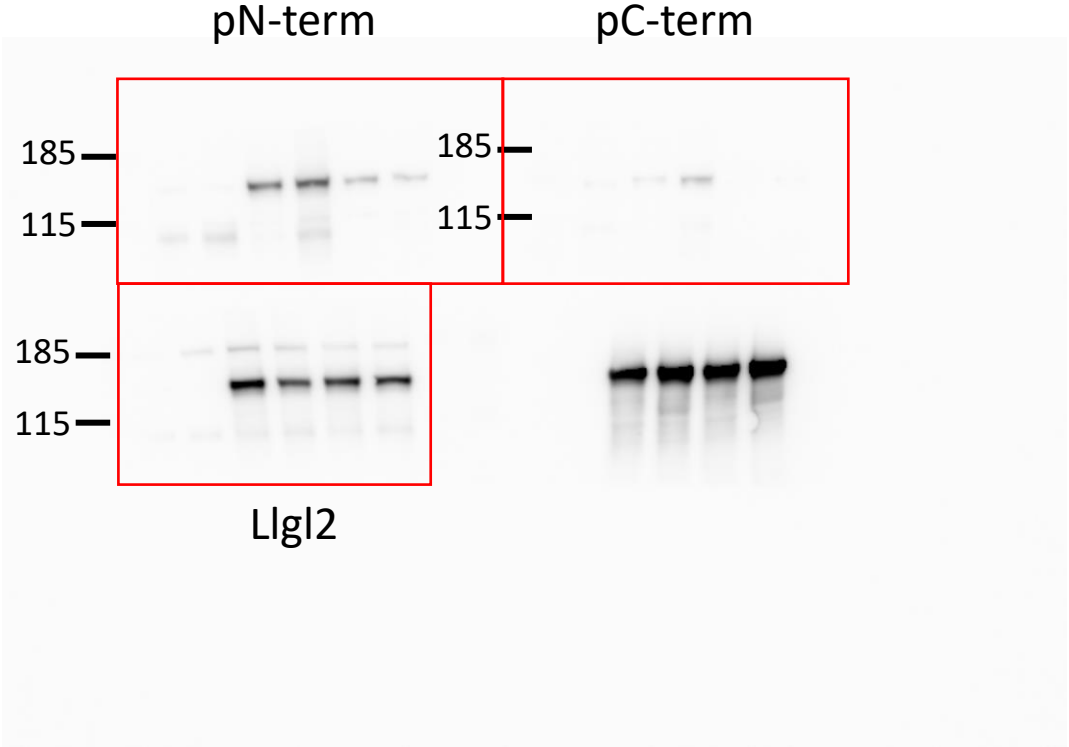

Supplement: Supplementary file 12 — Unprocessed western blots. [file 41594_2024_1425_MOESM12_ESM.pdf]

ED Fig 4i

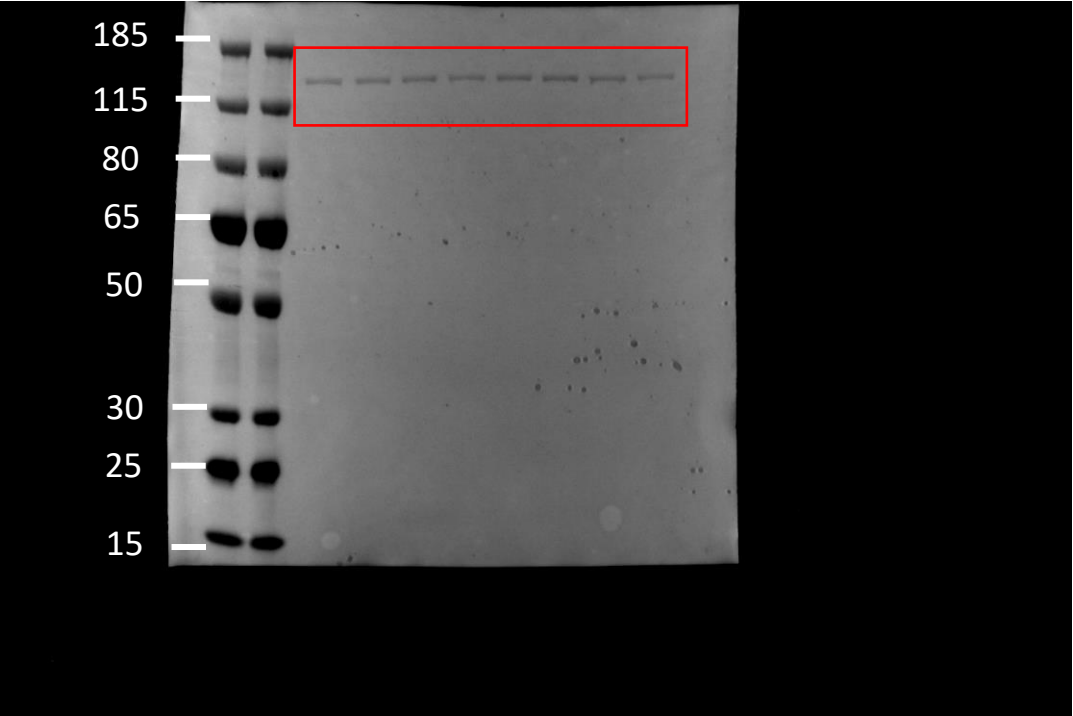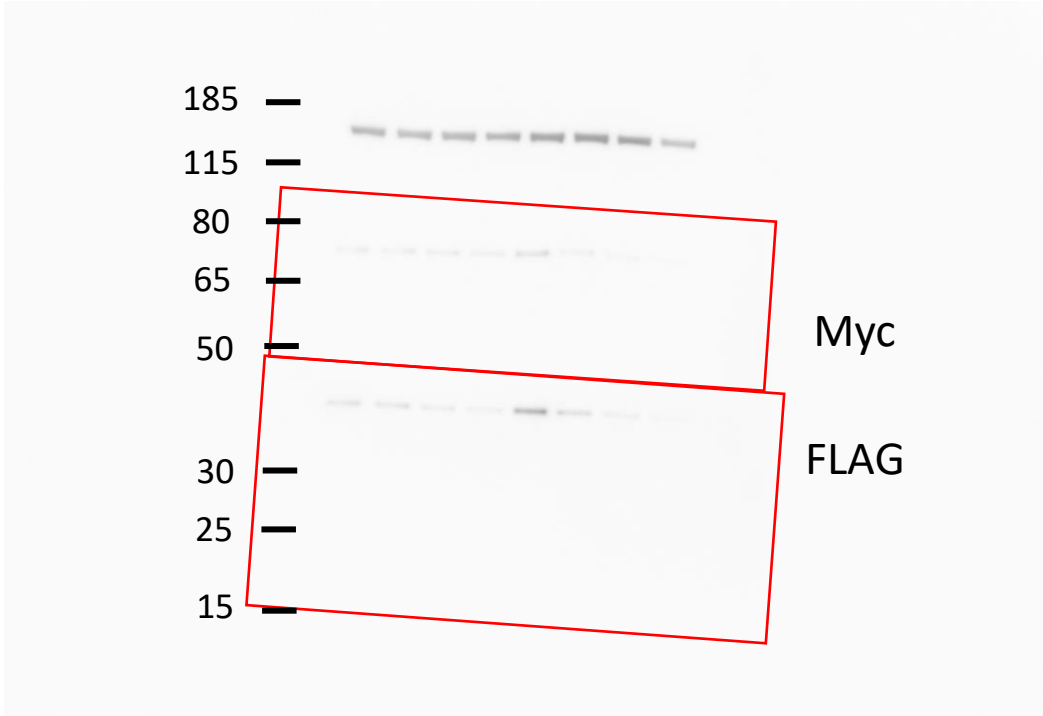

Supplement: Supplementary file 14 — Unprocessed western blots. [file 41594_2024_1425_MOESM14_ESM.pdf]
